# CRISPR screening reveals ZNF217 as a vulnerability in high-risk B-cell acute lymphoblastic leukemia

**DOI:** 10.7150/thno.100295

**Published:** 2025-02-18

**Authors:** Xi Qin, Keren Zhou, Lei Dong, Lu Yang, Wei Li, Zhenhua Chen, Chao Shen, Li Han, Yangchan Li, Anthony K.N. Chan, Sheela Pangeni Pokharel, Ying Qing, Meiling Chen, Kitty Wang, Keith Leung, Lillian Sau, Chun-Wei Chen, Xiaolan Deng, Rui Su, Jianjun Chen

**Affiliations:** 1 Department of Systems Biology, Beckman Research Institute of City of Hope, Duarte, CA 91010, USA.; 2 Center for RNA Biology and Therapeutics, Beckman Research Institute of City of Hope, Duarte, CA 91010, USA.; 3 Irell and Manella Graduate School of Biological Sciences, Beckman Research Institute of City of Hope, Duarte, CA 91010, USA.; 4 City of Hope Comprehensive Cancer Center, City of Hope, Duarte, CA 91010, USA.; 5 Gehr Family Center for Leukemia Research, City of Hope, Duarte, CA 91010, USA.; 6 Division of Epigenetic and Transcriptional Engineering, Beckman Research Institute, City of Hope, Duarte, CA 91010, USA.

**Keywords:** ZNF217, B-cell acute lymphoblastic leukemia (B-ALL), CoREST, FOS, CRISPR-Cas9 screen

## Abstract

**Rationale:** Despite substantial advancement in the treatment of B-cell acute lymphoblastic leukemia (B-ALL), it remains a leading cause of cancer mortality in children due to the high relapse rate. Moreover, the long-term survival rates for adult B-ALL patients are still less than 40%. The B-ALL patients carrying MLL rearrangements or BCR-ABL fusion represent high-risk B-ALL subtypes that face particularly dismal prognoses. This study aims to identify innovative therapeutic vulnerability for high-risk B-ALL.

**Methods:** The CRISPR-Cas9 screen was conducted to pinpoint genes essential for high-risk B-ALL cell survival/growth. Both *in vitro* and *in vivo* models were then employed to investigate the pathological role of ZNF217 in high-risk B-ALL. To characterize the downstream functionally essential targets of ZNF217, we performed RNA-seq and CUT&RUN-seq, followed by integrative bioinformatics analysis and experimental validation.

**Results:** Through the focused CRISPR-Cas9 screening, *ZNF217* emerged as the most essential gene for the cell survival/growth of B-ALL driven by MLL rearrangement or BCR-ABL. Through *in vitro* gain- and loss-of-function assays, we demonstrated that ZNF217 is indeed required for B-ALL cell survival/growth. Moreover, we established the B-ALL xenograft model and patient-derived xenograft (PDX) model and demonstrated that *ZNF217* depletion significantly suppressed B-ALL progression and substantially extended the survival of recipient mice. Through integrative multiple-omics analysis, we elucidated that ZNF217 exerts its oncogenic role in B-ALL through both CoREST-dependent and CoREST-independent mechanisms. Furthermore, we characterized FOS as a functionally essential downstream target of ZNF217, and ZNF217 inhibited FOS expression in a CoREST-independent manner.

**Conclusions:** Our findings highlight ZNF217 as a promising therapeutic target for the treatment of high-risk B-ALL, such as those carrying MLL-rearrangements or BCR-ABL fusion.

## Introduction

B-cell acute lymphoblastic leukemia (B-ALL), a type of malignancy of B lymphocyte progenitors 1, predominantly occurring around age 2-5 2, 3, with a second peak of incidence around age 50 2. It stands as the most common pediatric cancer, with a 5-year overall survival (OS) rate above 90% under contemporary therapies 3, 4. However, the prognosis for ALL worsens with age, with adults (age ≥ 40 years) achieving cure rates of only 30-40% 5, 6. This relatively poorer outcome observed in adult B-ALL compared to childhood cases is partly attributed to the higher occurrence of high-risk molecular lesions, such as BCR-ABL1 (30-40% in adults *vs.* 2-5% in children) and MLL rearrangements (MLL-r, 10-20% in adults *vs.* 5-7% in children) 7. Moreover, the outcome of childhood B-ALL patients with MLL-r or BCR-ABL1 remains dismal, with 5-year overall survival < 35% 6, 8. MLL-r and BCR-ABL1 usually lead to B-ALL relapse or refractory 9-11, giving rise to a much poorer prognosis. Therefore, there is an urgent need to identify innovative therapeutic strategies for the treatment of high-risk B-ALL, such as those driven by MLL-r or BCR-ABL.

Normal B cell development requires tightly orchestrated regulation of epigenetic modifications and transcription factors. Disturbances of B cell development can lead to diseases ranging from benign lymphoproliferation to malignant leukemia, such as B-ALL. Interestingly, alterations in epigenetic regulators have been identified in genome and exome sequencing of chemotherapy-resistant and high-risk subsets of *de novo* ALL 12. Additionally, two-thirds of mutations enriched in relapsed B-ALL occur in genes encoding epigenetic regulators 13, 14, underscoring the crucial role of epigenetic regulation in B-ALL pathogenesis. Moreover, emerging evidence suggests that RNA modification, such as *N*^6^-methyladenosine (m^6^A), plays a crucial role in early B cell development. For instance, m^6^A-dependent RNA decay mediated by YTHDF2 is critical for the transition from pro-B stage to large pre-B stage 15. Depletion of the m^6^A methyltransferase METTL14 severely impedes the transition from large pre-B stage to small pre-B stage 15. Blocking early B cell development at these two key transitions predisposes cells to malignant transformation into B-ALL 16-21, highlighting the implication of m^6^A modification in B-ALL leukemogenesis. Given the critical roles of epigenetic and epitranscriptomic modifications in B-ALL leukemogenesis, it remains elusive which regulator(s) of these modifications represent promising vulnerabilities for B-ALL therapy.

The zinc finger protein 217 (*ZNF217*) has emerged as a potential oncogene across a broad spectrum of cancers, including brain, breast, liver, lung, and uterine cancers 22-33. *ZNF217* amplification and overexpression are common occurrences in these malignancies, where it has been shown to promote cancer cell proliferation, survival, stemness, immortalization, metastasis, and drug resistance 27, 31, 33-39. Recent studies have highlighted ZNF217's role in modulating m^6^A RNA methylation, showcasing its diverse mechanisms of action, from blocking the m^6^A "writer" METTL3 in embryonic stem cells and breast cancer cells to enhancing the transcription of the m^6^A "eraser" FTO in adipocytes 40-45. Beyond its role as an m^6^A regulator, ZNF217 has been shown to function as a DNA-binding protein, regulating gene expression by altering histone configurations at target loci 46-48. It does so through recruiting specific histone-modifying proteins or complexes, often involving one or more components of the core CoREST complex (CoREST, LSD1, HDAC1/HDAC2) 48-52. However, the role and mechanism of ZNF217 in B-ALL remain unknown.

In the present study, we employed a focused CRISPR-Cas9-based screen targeting well-characterized genes associated with epigenetic and epitranscriptomic modifications in B-ALL models driven by MLL-r and BCR-ABL. This screen identified *ZNF217* as the top gene essential for B-ALL cell survival. Functionally, depletion of *ZNF217* significantly suppressed B-ALL cell growth/proliferation *in vitro* and notably prolonged the survival of B-ALL mice *in vivo*. Mechanistically, the oncogenic role of ZNF217 in B-ALL is not involved in the regulation of m^6^A modification. Instead, ZNF217 regulates its target genes through both CoREST-dependent and CoREST-independent mechanisms, relying on its DNA-binding capability. Collectively, our study underscores the crucial oncogenic role of ZNF217 in B-ALL and unveils novel insights into the molecular mechanisms underlying the pathogenesis of high-risk B-ALL, paving the way for the development of more effective targeted therapies for high-risk B-ALL.

## Results

### CRISPR library screen focusing on RNA/DNA epigenetics uncovers ZNF217 as a novel vulnerability in B-ALL

**Figure 1:**
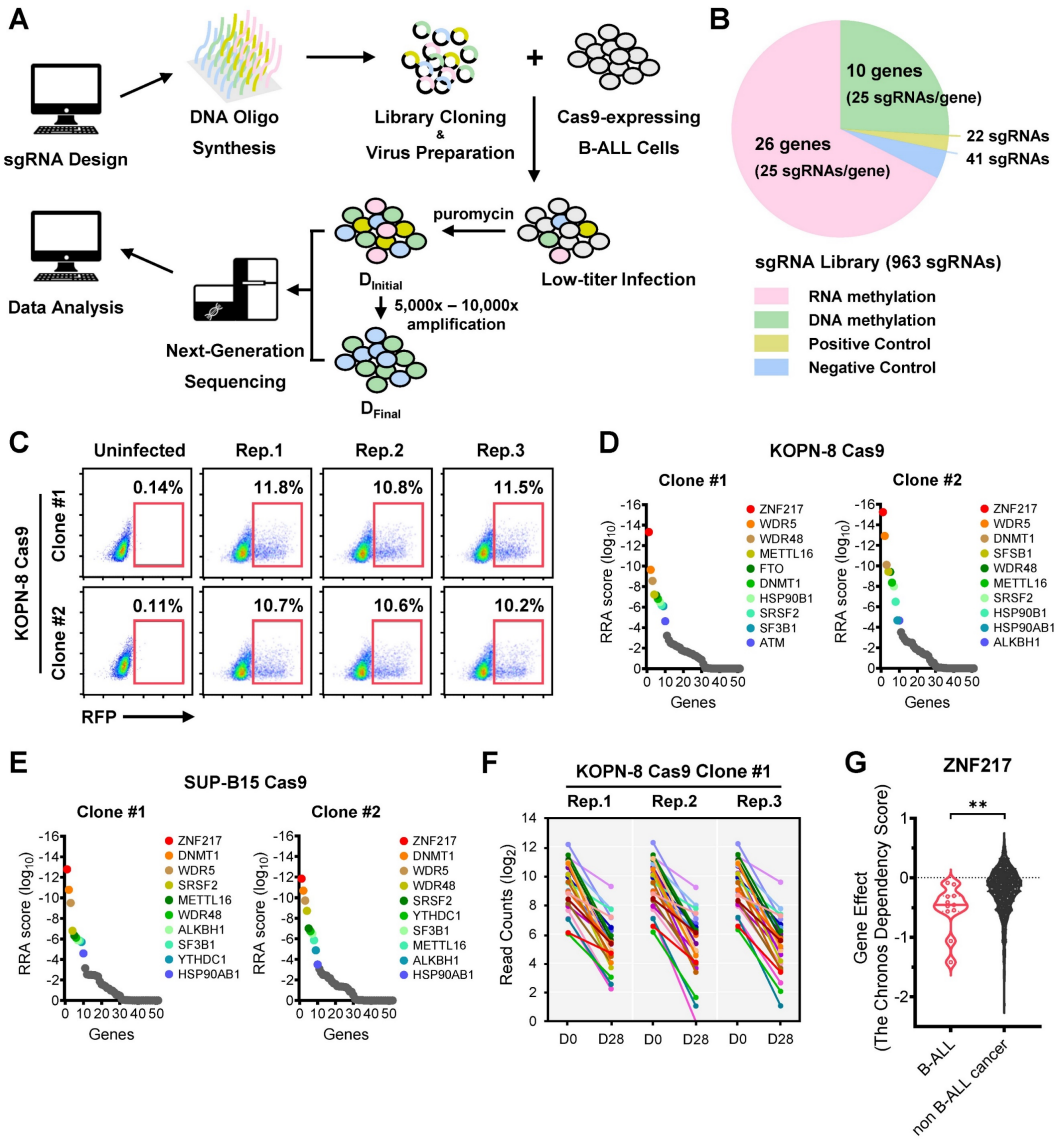
** CRISPR screening identifies ZNF217 as an essential gene for the survival of high-risk B-ALL cells. (A)** CRISPR screen workflow with KOPN-8 and SUP-B15 B-ALL cells. **(B)** Composition of the sgRNA library for CRISPR screening. **(C)** The lentiviral transduction efficiency of sgRNA library in CRISPR screening with KOPN-8 Cas9 single clones. **(D)** Negative selection ranks and RRA (robust ranking aggregation) scores in the two KOPN-8 Cas9 single clones. **(E)** Negative selection ranks and RRA scores in the two SUP-B15 Cas9 single clones. **(F)** Read counts of the 25 sgRNAs targeting *ZNF217* in KOPN-8 Cas9 Clone #1. **(G)** The Chronos Dependency Score of ZNF217 in B-ALL cell lines and non B-ALL cancer cell lines. The raw data was derived from the DepMap portal (https://depmap.org/portal/). n = 11 for B-ALL cell lines; n = 1,139 for non-B-ALL cancer cell lines. The p values were calculated using two-tailed *t*-test. ** p < 0.01.

To characterize the RNA/DNA epigenetics-related genes crucial for the growth and survival of B-ALL cells, we designed a specialized CRISPR library targeting 36 RNA and DNA methylation machinery-associated genes with 25 sgRNAs per gene (Figure 1A-1B; 900 sgRNAs in total). Additionally, we integrated a set of 41 negative-control sgRNAs targeting scrambled sequences or non-human genes such as firefly and renilla luciferase genes, along with 22 positive-control sgRNAs targeting common essential genes such as *MYC* and *BRD4* (Figure 1B and Supplementary Table 1). This library was lentivirally introduced into Cas9-expressing single clones of two human B-ALL cell lines, KOPN-8 and SUP-B15, which carry the *MLL-ENL* fusion gene and *BCR-ABL1* fusion gene, respectively (Figure 1C and Figure S1A). We incorporated 2 Cas9 single clones for each B-ALL cell line and 3 biological replicates for each Cas9 single clone. These cells were cultured until their amplification reached a range of 5,000 × to 10,000 ×, allowing for the sufficient elimination of cells in which essential genes were disrupted. The frequency of each integrated sgRNA construct at the “initial point” and “final point” of amplification was assessed using high-throughput sequencing (Figure 1A and Figure S1B-S1E).

Validating the efficacy of our screens, we noted a significant depletion of positive control sgRNAs targeting common essential genes, whereas those negative control sgRNAs were not depleted in the cell population during proliferation/amplification (Figure S1F-S1G). Moreover, we observed that sgRNAs targeting several RNA and DNA methylation machinery-associated genes, such as *WDR5*, *METTL16*, and *ZNF217*, were substantially depleted during the screens (Figure S1F-S1G). Employing the Model-based Analysis of Genome-wide CRISPR-Cas9 Knockout (MAGeCK) algorithm, our focused CRISPR screen revealed *ZNF217* (encoding Zinc Finger Protein 217) as the top-ranked essential gene in both KOPN-8 and SUP-B15 B-ALL cells (Figure 1D-1F and Figure S1H-S1J). Furthermore, we analyzed the Cancer Dependency Map (DepMap), a genome-scale CRISPR-Cas9 knockout (KO) for 19,144 genes across 1,139 cancer cell lines. Our findings indicated that ZNF217 played a more critical role in the survival of B-ALL cell lines compared to non-B-ALL cancer cell lines (Figure 1G). Moreover, we found that *ZNF217* is significantly overexpressed across multiple B-ALL subtypes, including high-risk B-ALL driven by MLL-r or BCR-ABL, relative to healthy controls (Figure S1K). Collectively, our focused CRISPR screening, along with the DepMap and ZNF217 expression results, underscores the indispensable role of ZNF217 in the survival/proliferation of human B-ALL cells.

### ZNF217 promotes B-ALL cell growth and survival *in vitro*

**Figure 2:**
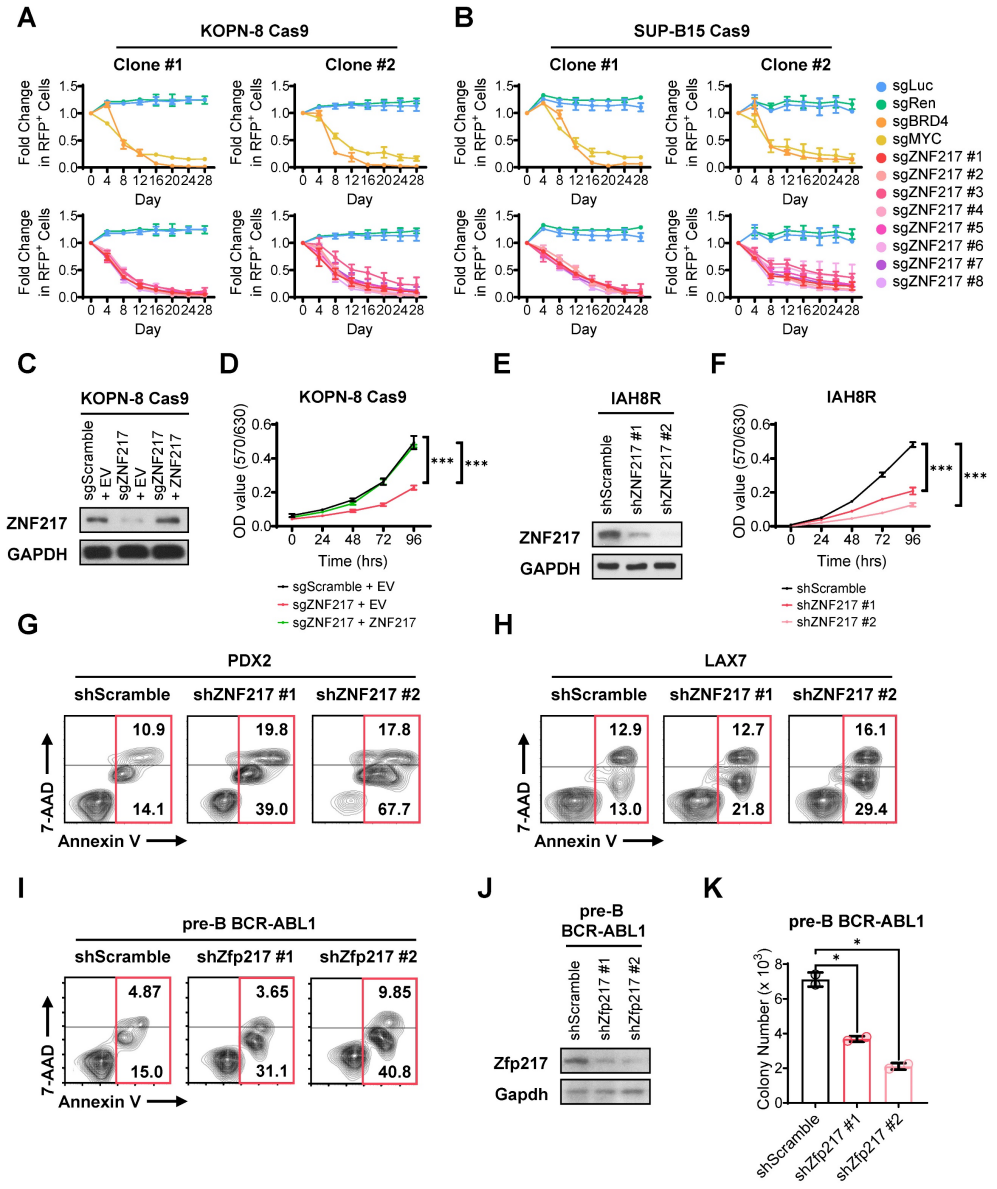
***ZNF217* depletion significantly inhibits proliferation and induces apoptosis of B-ALL cells *in vitro*. (A)** Effect of *ZNF217* KO on the fitness/growth of KOPN-8 B-ALL cells as determined by growth competition assay. The sgRNAs targeting *MYC* and *BRD4* were included as positive controls, while the sgRNAs targeting firefly and renilla luciferase genes were included as negative controls. Data was presented as mean ± SD (n = 3 biological replicates). **(B)** Effect of *ZNF217* KO on the fitness/growth of SUP-B15 B-ALL cells as determined by growth competition assay. Data was presented as mean ± SD (n = 3 biological replicates). **(C)** *ZNF217* KO and overexpression efficiency in KOPN-8 Cas9 cells as determined by western blotting. **(D)** Effect of *ZNF217* KO and rescued expression on the growth of KOPN-8 cells as determined by MTT assay. Data was presented as mean ± SD (n = 4 biological replicates). **(E)** *ZNF217* KD efficiency in IAH8R B-ALL PDX cells as determined by western blotting. **(F)** Effect of *ZNF217* KD on the growth of IAH8R B-ALL PDX cells as determined by MTT assay. Data was presented as mean ± SD (n = 4 biological replicates). **(G)** Effect of *ZNF217* KD on the apoptosis of PDX2 B-ALL PDX cells. **(H)** Effect of *ZNF217* KD on the apoptosis of LAX7 B-ALL PDX cells. **(I)** Effect of *Zfp217* KD on the apoptosis of murine pre-B-ALL cells driven by BCR-ABL1. **(J)** *Znf217* KD efficiency in pre-B-ALL cells driven by BCR-ABL1 as determined by western blotting. **(K)** Effect of *Zfp217* KD on the colony-forming ability of murine pre-B-ALL cells driven by BCR-ABL1. Data was presented as mean ± SD (n = 2 biological replicates). The p values were calculated using a two-tailed *t*-test. * p < 0.05; *** p < 0.001.

To validate our CRISPR screening findings, we performed a growth competition assay in both KOPN-8 and SUP-B15 B-ALL cells. In this assay, we utilized 8 sgRNAs targeting *ZNF217*, along with 2 positive-control sgRNAs targeting essential genes and 2 negative-control sgRNAs targeting non-essential sequences, all sourced from the sgRNA library employed in the CRISPR screen. As an indicator for transduction-positive cells, we co-expressed RFP with the sgRNA. Our results revealed a significant decrease in the competitive fitness of B-ALL cells carrying sgRNAs targeting *ZNF217* compared to their non-targeted counterparts, resembling the trends observed with cells harboring sgRNA targeting common essential genes (*MYC* and *BRD4*; Figure 2A-2B and Figure S2A-S2B). As a control, cells with sgRNA targeting non-essential sequences retained their competitive fitness (Figure 2A-2B). Through an independent MTT assay, we demonstrated that KO of *ZNF217* markedly impeded the growth of B-ALL cells, which could be fully reversed by overexpression of *ZNF217* (Figure 2C-2D), underscoring the high specificity of the *ZNF217* sgRNA.

To further elucidate the pathological roles of ZNF217, we conducted both loss-of-function and gain-of-function studies with human B-ALL cell lines, patient-derived xenograft (PDX) cells, and murine B-ALL cells. As expected, *ZNF217* knockdown (KD) using short hairpin RNAs (shRNAs) also resulted in profound growth inhibition in both human B-ALL PDX cells (Figure 2E-2F and Figure S2C-S2F) and cell lines (Figure S2G-S2J), akin to the effects observed with CRISPR KO. Additionally, *ZNF217* KD induced remarkable apoptosis in B-ALL cells (Figure 2G-2I and Figure S2K-S2L). Conversely, the ectopic expression of ZNF217 significantly enhanced human B-ALL cell survival/growth (Figures S2M-S2P). Furthermore, we noted that depletion of *Zfp217*, the murine homolog of *ZNF217*, in murine B-ALL cells severely affected colony formation (Figure 2J-2K), highlighting the essential role of ZNF217 in B-ALL cell repopulation. Overall, our findings corroborate the oncogenic role of ZNF217 in promoting B-ALL cell survival/growth and repopulation, while suppressing apoptosis.

### ZNF217 promotes B-ALL maintenance and progression *in vivo*

**Figure 3:**
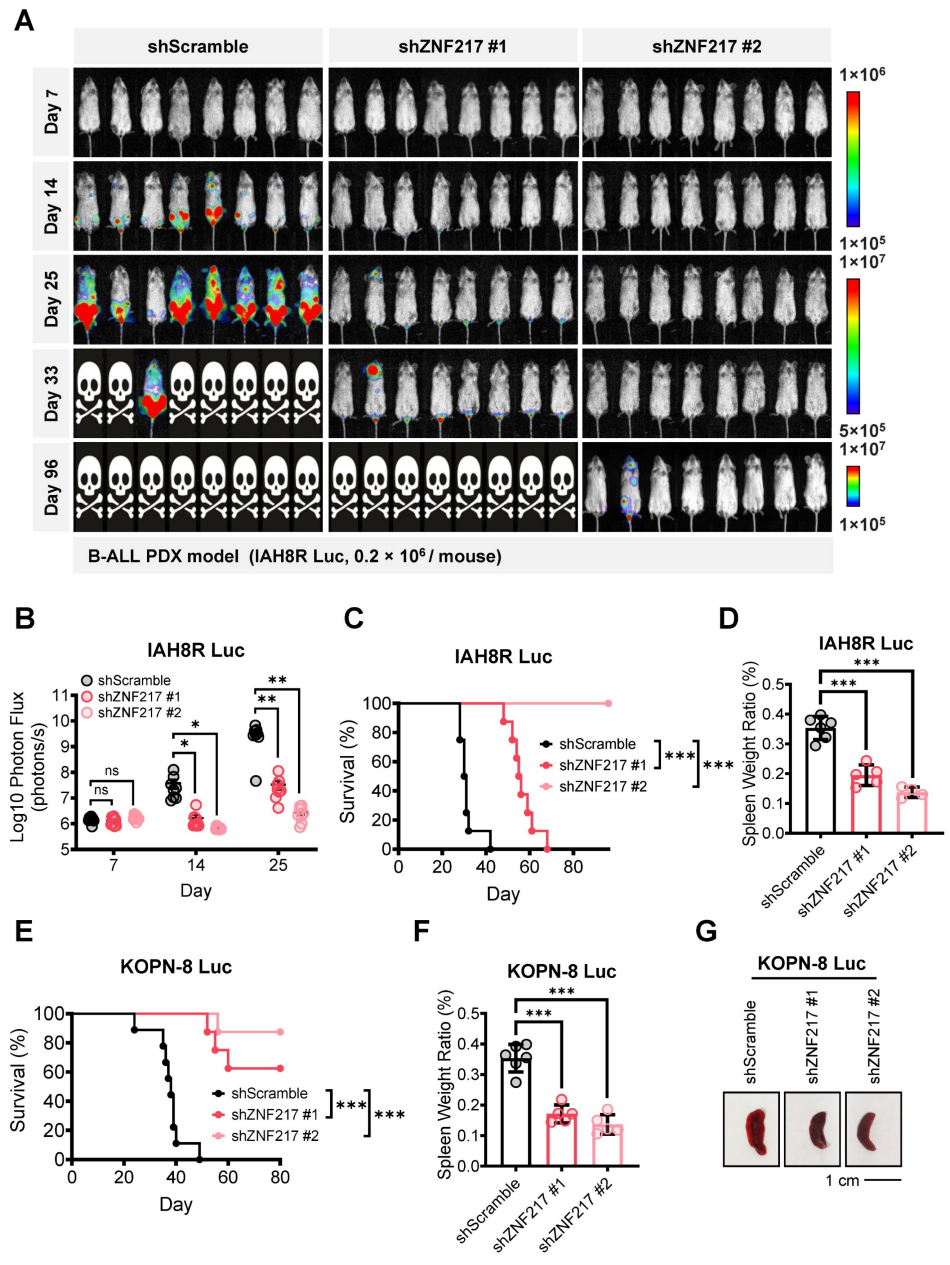
***ZNF217* depletion significantly suppresses B-ALL progression and extends overall survival in mice. (A)** Effect of *ZNF217* KD on leukemia burden in B-ALL PDX receipt (NSG) mice, as determined by bioluminescence imaging. **(B)** Quantification of bioluminescence signals in B-ALL PDX receipt mice. n = 8 for each group. **(C)** Effect of *ZNF217* KD on overall survival in B-ALL PDX receipt mice. n = 8 for each group. **(D)** Effect of *ZNF217* KD on splenomegaly in B-ALL PDX receipt mice. Data was presented as mean ± SD (n = 6 for shScramble; n = 5 for shZNF217 #1; n = 4 for shZNF217 #2). **(E)** Effect of *ZNF217* KD on overall survival of NSG mice in B-ALL xenograft receipt mice. n = 9 for shScramble; n = 8 for shZNF217 #1; n = 8 for shZNF217 #2. **(F)** Effect of *ZNF217* KD on splenomegaly in B-ALL xenograft receipt mice. Data was presented as mean ± SD (n = 6 for shScramble; n = 5 for shZNF217 #1; n = 5 for shZNF217 #2). **(G)** Representative spleen images of B-ALL xenograft receipt mice. The p values were calculated using two-tailed *t*-test (B, D, and F) and log-rank test (C and E). ns, not significant; * p < 0.05; ** p < 0.01; *** p < 0.001.

We then established B-ALL 'human-in-mouse' xenograft and PDX models to study the role of ZNF217 in B-ALL maintenance and progression *in vivo*. To closely monitor B-ALL cell infiltration and leukemia progression, we labeled both B-ALL PDX cells (IAH8R) and cell line (KOPN-8) with a firefly luciferase gene before transplanting them into NSG recipients via tail vein injection (Figure S3A-S3F). Employing bioluminescence imaging to track leukemia burden, we observed that *ZNF217* depletion significantly suppressed B-ALL progression in both the PDX model (Figure 3A-3B) and the xenograft model (Figure S3G). Consistent with these findings, depletion of *ZNF217* significantly extended the overall survival of recipient NSG mice (Figure 3C and 3E) and inhibited splenomegaly (Figure 3D, 3F, 3G). Thus, our studies demonstrated the critical role of ZNF217 in the maintenance and progression of human B-ALL *in vivo*.

### ZNF217 is involved in B-ALL independent of m^6^A-associated mechanisms

ZNF217 has been implicated in modulating m^6^A RNA methylation through diverse mechanisms. For instance, ZNF217 was reported to reduce mRNA m^6^A methylation in embryonic stem cells and breast cancer cells by sequestering the m^6^A 'writer' METTL3, thereby supporting embryonic stem cell pluripotency and promoting breast cancer progression 40-43. Additionally, ZNF217 was also reported to suppress mRNA m^6^A methylation by directly activating the transcription of m^6^A 'eraser' FTO in adipocytes and nucleus pulposus cells, regulating adipogenesis and intervertebral disc degeneration, respectively 44, 45. Furthermore, in adipocytes, ZNF217 interacts with m^6^A 'reader' YTHDF2, which is essential for maintaining the demethylation activity of FTO 44. These studies prompted us to investigate whether ZNF217 influences B-ALL through m^6^A-associated mechanisms. We first conducted exogenous and endogenous co-IP experiments to explore the potential direct interaction between ZNF217 and m^6^A machinery in B-ALL cells. Our results revealed that ZNF217 does not interplay with either METTL3 or YTHDF2 in human B-ALL (KOPN-8 and SUP-B15) cells (Figure S4A-S4C). Reciprocal co-IP further confirmed the absence of interaction with METTL3 in KOPN-8 cells (Figure S4D). Additionally, ZNF217 does not interact with other RNA methylation machinery-associated proteins such as METTL14, METTL16, IGF2BP1, IGF2BP2, and PCIF1 (Figure S4A). We then evaluated whether ZNF217 promoted the transcription of FTO in B-ALL cells. Our quantitative real-time PCR data indicated that neither the depletion nor overexpression of *ZNF217* significantly altered *FTO* mRNA levels in KOPN-8 cells (Figure S4E-S4H), suggesting that ZNF217 does not regulate *FTO* transcription in B-ALL cells. Finally, we conducted dot blot assay and independent ultra-high-pressure liquid chromatography coupled with triple-quadrupole tandem mass spectrometry (UHPLC-QQQ-MS/MS) assay to examine whether ZNF217 impacts global m^6^A levels in B-ALL cells. Our results demonstrated that neither the depletion nor overexpression of *ZNF217* significantly altered global m^6^A levels in total RNA or poly(A)^+^ mRNA in KOPN-8 cells (Figure S4I-S4M). Taken together, our results suggest that ZNF217's oncogenic role in B-ALL is unlikely to be mediated through m^6^A-associated mechanisms.

### ZNF217 interacts with the CoREST complex to mediate histone modifications in B-ALL cells

**Figure 4:**
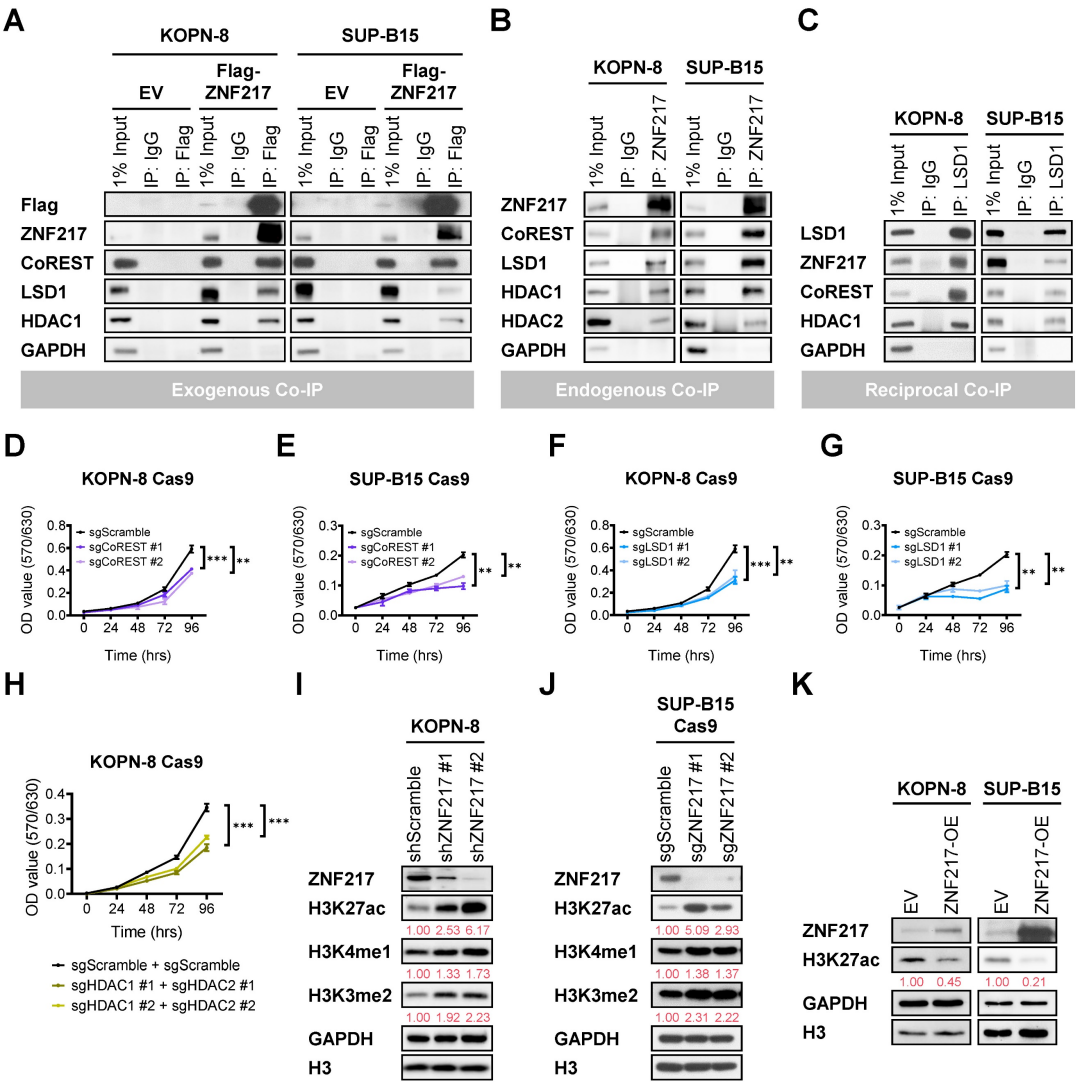
** ZNF217 interacts with the CoREST complex to mediate histone modifications in B-ALL cells. (A)** Exogenous co-IP assay using KOPN-8 and SUP-B15 cells with ectopic expression of Flag-tagged ZNF217 or an empty vector. **(B)** Endogenous co-IP assay using KOPN-8 and SUP-B15 cells. **(C)** Reciprocal co-IP assay using KOPN-8 and SUP-B15 cells. **(D)** Effect of *CoREST* KO on the growth of KOPN-8 cells as determined by MTT assay. Data was presented as mean ± SD (n = 4 biological replicates). **(E)** Effect of *CoREST* KO on the growth of SUP-B15 cells as determined by MTT assay. Data was presented as mean ± SD (n = 4 biological replicates). **(F)** Effect of *LSD1* KO on the growth of KOPN-8 cells as determined by MTT assay. Data was presented as mean ± SD (n = 4 biological replicates). **(G)** Effect of *LSD1* KO on the growth of SUP-B15 cells as determined by MTT assay. Data was presented as mean ± SD (n = 4 biological replicates). **(H)** Effect of *HDAC1/HDAC2* double KO on the growth of KOPN-8 cells as determined by MTT assay. Data was presented as mean ± SD (n = 4 biological replicates). **(I)** *ZNF217* KD efficiency in KOPN-8 cells and the effect on H3K27ac deacetylation, H3K4me1 demethylation, and H3K4me2 demethylation, as determined by Western blotting. Quantification of H3K27ac, H3K4me1, and H3K4me2 levels is shown in red beneath the corresponding Western blot bands. **(J)** *ZNF217* KO efficiency in SUP-B15 Cas9 cells and the effect on H3K27ac deacetylation, H3K4me1 demethylation, and H3K4me2 demethylation, as determined by Western blotting. Quantification of H3K27ac, H3K4me1, and H3K4me2 levels is shown in red beneath the corresponding Western blot bands. **(K)** *ZNF217* OE efficiency in KOPN-8 and SUP-B15 cells and the effect on H3K27ac deacetylation as determined by Western blotting. Quantification of H3K27ac levels is shown in red beneath the corresponding Western blot bands. The p values were calculated using a two-tailed *t*-test. ** p < 0.01; *** p < 0.001.

ZNF217 has also been reported to function as a DNA-binding protein, modulating the expression of its target genes by modifying histone structures at their loci 26, 46-48, 51, 52. The CoREST (Co-repressor of Repressor Element 1 Silencing Transcription factor) protein is a component of the CoREST complex, which includes histone deacetylase 1 (HDAC1) or its close paralog HDAC2, the scaffolding protein CoREST, and lysine-specific demethylase 1 (LSD1). ZNF217 has been identified as a critical component of the CoREST complex by recruiting the core CoREST complex, CoREST, LSD1, and HDAC1/HDAC2 26, 46, 48-54. In addition, we found that CoREST and LSD1 exhibit highly significant co-dependency with ZNF217 in genome-wide CRISPR KO screens across 1,139 cancer cell lines (Figure S5A and Supplementary Table 2). Therefore, we conducted co-IP assays to explore the potential interactions between ZNF217 and CoREST complex components in B-ALL cells. Our co-IP assays revealed direct interactions between either ectopic expressed or endogenous ZNF217 and the core components of the CoREST complex, including CoREST, LSD1, HDAC1, and HDAC2, in both KOPN-8 and SUP-B15 cells (Figure 4A-4B). This identification was further confirmed by reciprocal co-IP assays in B-ALL cells (Figure 4C). Moreover, KO of *CoREST, LSD1*, or *HDAC1* plus *HDAC2* phenocopied the growth-inhibitory effects caused by *ZNF217* depletion in B-ALL cells (Figure 4D-4H and Figure S5B-S5D).

The CoREST complex acts as a transcriptional corepressor, facilitating H3K4me1 and H3K4me2 demethylation by LSD1 and histone deacetylation by HDAC1 or HDAC2 49, 50, 53-59. In B-ALL cells, we employed CRISPR-Cas9 to achieve double KO of *HDAC1/HDAC2* and validated the deacetylation activities of HDAC1/2 on H3K27ac (Figure S5D). Furthermore, we investigated the impacts of *ZNF217* depletion on H3K4me1, H3K4me2, and H3K27ac modifications in KOPN-8 and SUP-B15 B-ALL cells. Our Western blotting analysis revealed a substantial increase in H3K27ac, H3K4me1, and H3K4me2 levels upon *ZNF217* KD (Figure 4I) or KO (Figure 4J). Conversely, forced expression of ZNF217 led to decreased H3K27ac levels in B-ALL cells (Figure 4K). These findings collectively support the conclusion that ZNF217 modulates histone modifications in B-ALL cells through its interaction with the CoREST complex, which contributes, at least partially, to its oncogenic functions in B-ALL.

Additionally, we analyzed genome-wide CRISPR screen datasets from DepMap and observed that the dependency of B-ALL cells on CoREST and HDAC1 mirrors their dependency on ZNF217 (Figure S5E). Although LSD1 also contributes to the survival and growth of B-ALL cells, its role appears less critical than that of ZNF217 (Figure S5E). HDAC2 does not play a crucial role in the survival and growth of B-ALL cells (Figure S5E), likely functioning as a redundant counterpart to HDAC1. These findings align with our observations regarding the effects of *ZNF217* depletion on histone modifications. Specifically, *ZNF217* depletion in B-ALL cells caused a more pronounced increase in H3K27ac levels than in H3K4me1 and H3K4me2 levels (Figure 4I-4J), suggesting that the regulation of H3K27ac may be more central to ZNF217's oncogenic role than does the regulation of H3K4me1 and H3K4me2. Overall, B-ALL cells exhibit a higher dependency on ZNF217 than on the CoREST complex members (Figure S5E), indicating that ZNF217's oncogenic functions in B-ALL may extend beyond its interaction with the CoREST complex.

### CRISPR gene tiling screen reveals functional domains of ZNF217 in B-ALL

**Figure 5:**
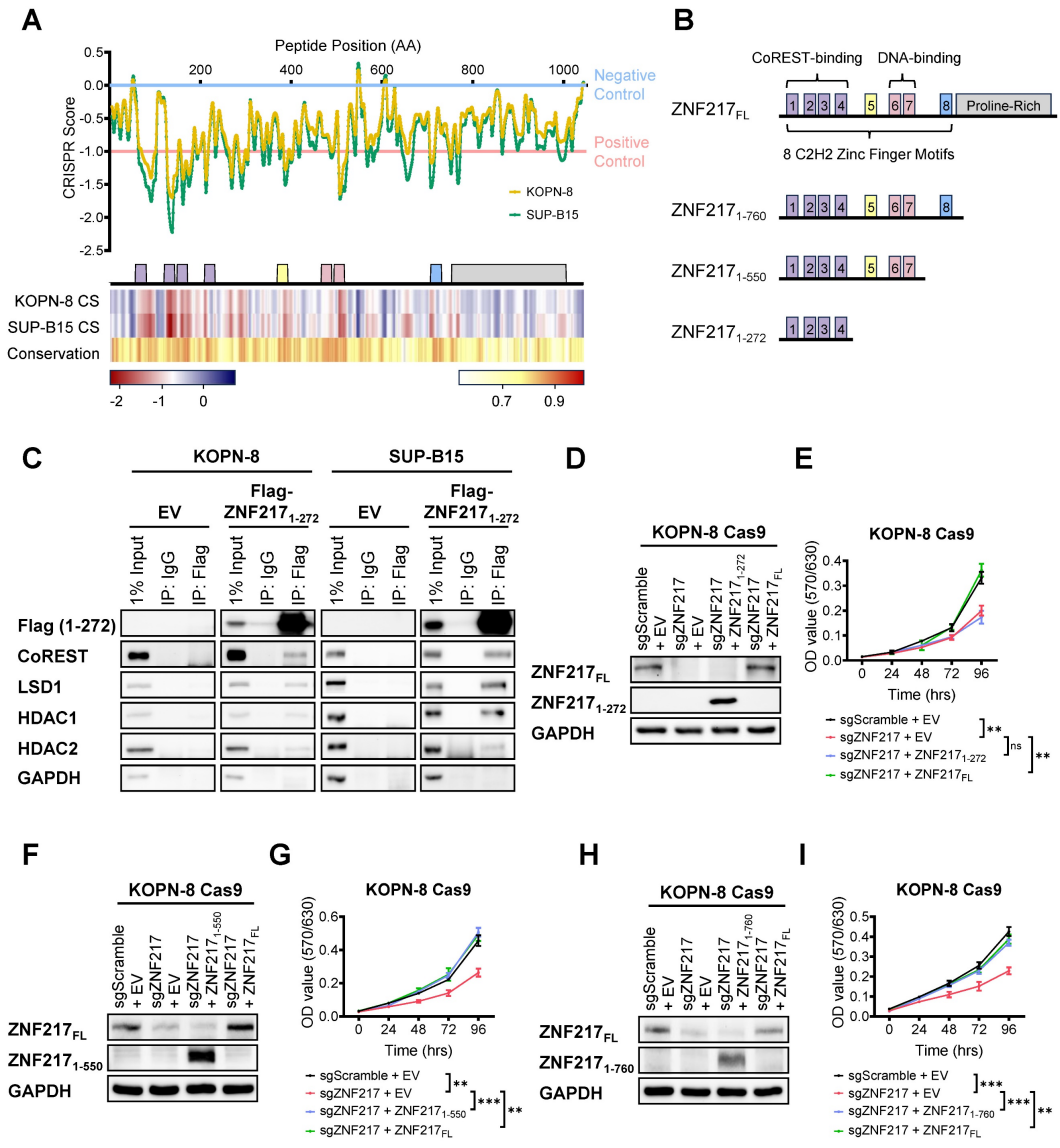
** CRISPR gene-tiling screen and ZNF217 truncation studies identify functionally important domains of ZNF217 in B-ALL. (A)** 2D annotation of the smoothened CRISPR scores of the 416 *ZNF217* sgRNAs in KOPN-8 Cas9 and SUP-B15 Cas9 single clones was shown in the upper panel. The CRISPR scores have been normalized against the median scores of the negative control sgRNAs (set at 0.0) and the positive control sgRNAs (set at -1.0). Heatmaps of the smoothened CRISPR scores and conservation scores were shown in the lower panel. **(B)** A scheme of ZNF217 truncation constructs. **(C)** Exogenous Co-IP assay using KOPN-8 and SUP-B15 cells with ectopic expression of Flag-tagged ZNF217_1-272_ truncation or an empty vector. **(D)** *ZNF217* KO, ZNF217_1-272_ truncation OE, and full-length ZNF217 OE efficiency in KOPN-8 Cas9 cells as determined by Western blotting. **(E)** Effect of *ZNF217* KO, ZNF217_1-272_ restored expression, and full-length ZNF217 restored expression on the growth of KOPN-8 cells, as determined by MTT assay. Data was presented as mean ± SD (n = 4 biological replicates). **(F)** *ZNF217* KO, ZNF217_1-550_ truncation OE, and full-length ZNF217 OE efficiency in KOPN-8 Cas9 cells as determined by Western blotting. **(G)** Effect of *ZNF217* KO, ZNF217_1-550_ restored expression, and full-length ZNF217 restored expression on the growth of KOPN-8 cells, as determined by MTT assay. Data was presented as mean ± SD (n = 4 biological replicates). **(H)** *ZNF217* KO, ZNF217_1-760_ truncation OE, and full-length ZNF217 OE efficiency in KOPN-8 Cas9 cells as determined by Western blotting. **(I)** Effect of *ZNF217* KO, ZNF217_1-760_ restored expression, and full-length ZNF217 restored expression on the growth of KOPN-8 cells, as determined by MTT assay. Data was presented as mean ± SD (n = 4 biological replicates). The p values were calculated using a two-tailed *t*-test. ns, not significant; * p < 0.05; ** p < 0.01; *** p < 0.001.

To gain further mechanistic insights into functional regions within ZNF217 that are crucial for B-ALL cell survival, we leveraged an unbiased high-density CRISPR gene tiling screen 60-66. This technique allows us to pinpoint the functionally essential domains within a specific protein through CRISPR-mediated mutagenesis. To achieve this objective, we designed a sgRNA library comprising 416 sgRNAs targeting every "NGG" protospacer adjacent motif within ZNF217's coding exons, which enabled saturation mutagenesis with an average density of 7.5 base pairs (or 2.5 amino acids) per sgRNA (Supplementary Table 1). Subsequently, this library was introduced into Cas9-expressing single clones of KOPN-8 and SUP-B15 through lentivirus transduction. Following lentivirus transduction, the frequencies of each sgRNA sequence were assessed at the “initial” and “final” points of amplification using NextSeq550 sequencing. After refining the CRISPR scores of each sgRNA using a locally smoothened model, we aligned the scores to the 2D structure of ZNF217 protein 67-69. Our CRISPR tiling screen unveiled the critical roles of multiple regions within the ZNF217 protein (Figure 5A upper panel). Overall, the N-terminal and middle regions, which encompass the 8 zinc finger motifs, were more essential for B-ALL cell survival, than does the C-terminal proline-rich domain. To evaluate whether the editing efficiency of individual *ZNF217* sgRNAs affects their CRISPR scores in our ZNF217 CRISPR tiling screen, we analyzed the correlation between the CRISPR scores of the 416 *ZNF217* sgRNAs and their predicted on-target efficacy scores. The on-target efficacy scores were estimated using the Genetic Perturbation Platform (Broad Institute) 70. Our analysis revealed no significant correlation between the on-target efficacy scores and the CRISPR scores of the sgRNAs (Figure S6A-S6B and Supplementary Table 3). This finding suggests that the ZNF217 CRISPR tiling pattern is not influenced by CRISPR editing efficiency.

Concurrently, we conducted a conservation analysis utilizing the Jensen-Shannon divergence-based method to predict the functionally important residues in ZNF217 protein sequence 71. This analysis spanned eight species: human, chimpanzee, rhesus monkey, horse, pig, cat, Chinese hamster, and mouse. Strikingly, the results of this conservation analysis were consistent with our CRISPR tiling screen findings, indicating a higher degree of conservation and essentiality in the zinc finger motifs compared to the C-terminal proline-rich domain (Figure 5A lower panel). This suggests the oncogenic role of ZNF217 in B-ALL might predominantly rely on these highly conserved zinc finger motifs. Previous research has identified zinc finger motifs ZF1-4 as crucial for CoREST protein interaction and ZF6-7 as key for DNA binding 51, 69, 72. Additionally, motifs adjacent to zinc finger motif ZF8 have been identified as pivotal for binding to the transcription co-repressor C-terminal binding protein (CtBP) 51, 73. Our ZNF217 CRISPR tiling screen also underscored the functional importance of the ZF5 zinc finger motif, which has not been reported previously.

To further elucidate the roles of ZNF217's zinc finger motifs in B-ALL, we generated a series of ZNF217 truncation constructs (Figure 5B). The ZNF217_1-760_ truncation encompassed all eight zinc finger motifs while excluding the C-terminal proline-rich domain. The ZNF217_1-550_ truncation included the first seven zinc finger motifs, and the ZNF217_1-272_ truncation was limited to the first four zinc finger motifs known to interact with the CoREST protein. We subsequently conducted Co-IP assays in KOPN-8 and SUP-B15 cells to explore the interaction between truncated ZNF217 proteins and the CoREST complex. Our results revealed that the ZNF217_1-272_ truncation is sufficient to interact with the CoREST protein as well as with LSD1, HDAC1, and HDAC2 in both B-ALL cell lines tested (Figure 5C). These findings corroborated previous reports that the ZF1-4 motifs bind to the CoREST protein 51. However, despite the interaction of these motifs with the CoREST complex in B-ALL cells, our MTT assays showed that, unlike the forced expression of full-length ZNF217, forced expression of the ZNF217_1-272_ truncation failed to rescue the growth inhibition caused by *ZNF217* KO (Figure 5D and 5E). This suggests that the interaction of ZNF217 protein with the CoREST complex alone is not sufficient to fulfill ZNF217's oncogenic functions in B-ALL. In contrast to the ZNF217_1-272_ truncation, forced expression of either the ZNF217_1-550_ or ZNF217_1-760_ truncations in B-ALL cells nearly completely reversed the growth inhibition caused by *ZNF217* KO, closely mirroring the effect of full-length ZNF217 expression (Figures 5F-5I). This indicates that the ZF5-7 motifs are necessary for ZNF217's overall oncogenic function.

In summary, while ZNF217 directly interacts with the CoREST complex in B-ALL, our findings suggest that its oncogenic functions in B-ALL extend beyond this interaction with the CoREST complex, likely involving both CoREST-dependent and -independent mechanisms. Furthermore, our CRISPR tiling screen highlights the functional significance of ZNF217's DNA-binding capacity in B-ALL. Additionally, we have identified ZF5, whose precise role remains unknown, as an essential zinc finger motif contributing to ZNF217's oncogenic effects in B-ALL. This unveils a previously unrecognized aspect of ZNF217's functionality.

### ZNF217 modulates the expression of its target genes through both CoREST-dependent and CoREST-independent mechanisms in B-ALL

**Figure 6:**
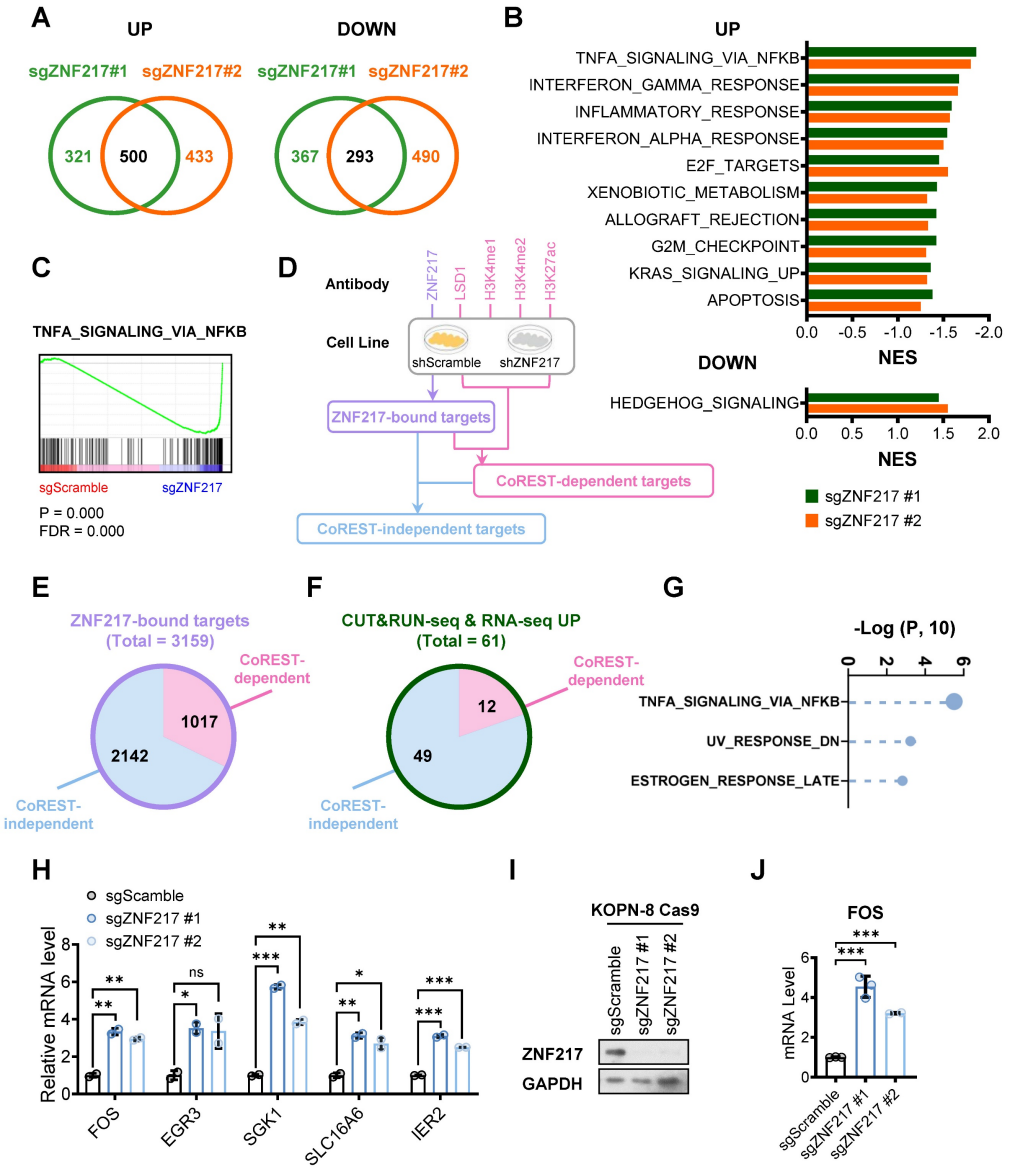
** RNA-seq and CUT&RUN-seq identify downstream targets of ZNF217 in B-ALL. (A)** Venn plot showing the overlap of genes upregulated (left panel) or downregulated (right panel) caused by *ZNF217* KO mediated by two independent sgRNAs targeting *ZNF217* in KOPN-8 Cas9 cells. **(B)** GSEA analysis showing the gene sets upregulated (upper panel) or downregulated (lower panel) in KOPN-8 cells upon *ZNF217* KO. **(C)** The GSEA enrichment plot of a representative upregulated gene set. **(D)** A workflow of CUT&RUN-seq sample preparation. **(E)** Pie chat of the 3,159 ZNF217-bound genes in KOPN-8 cells identified by CUT&RUN-seq. **(F)** The overlap of upregulated genes identified by RNA-seq and ZNF217-bound genes identified by CUT&RUN-seq. **(G)** GSEA analysis of the CoREST-independent targets upregulated upon *ZNF217* KO in KOPN-8 cells. **(H)** Expression fold-changes of the core-enriched genes in (G) as determined by RNA-seq. Data was presented as mean ± SD (n = 2 biological replicates). **(I)** *ZNF217* KO efficiency in KOPN-8 Cas9 cells as determined by western blotting. **(J)** Effect of *ZNF217* KO on FOS expression in KOPN-8 cells, as determined by RT-qPCR. Data was presented as mean ± SD (n = 3 technical replicates). The p values were calculated using a two-tailed *t*-test. ns, not significant; * p < 0.05; ** p < 0.01; *** p < 0.001.

To uncover the critical downstream targets of ZNF217, we performed a comprehensive multiple-omics analysis in B-ALL cells with or without *ZNF217* depletion. First, our transcriptome-wide RNA sequencing (RNA-seq) revealed 500 upregulated genes and 293 downregulated genes (fold change ≥ 2) upon *ZNF217* KO in KOPN-8 cells (Figure 6A). Gene set enrichment analysis (GSEA) showed that the upregulated genes were enriched in immune response, such as “TNFα signaling via NF-κΒ”, “interferon-γ response”, “inflammatory response”, and “interferon-α response”, as well as pathways related to cell proliferation and apoptosis, such as “TNFα signaling via NF-κΒ”, “E2F targets”, “G2M checkpoint”, and “apoptosis” (Figure 6B and 6C). The downregulated genes were predominantly involved in the hedgehog signaling pathway (Figure 6B). Next, we performed cleavage under targets and release using nuclease followed by next-generation sequencing (CUT&RUN-seq) to characterize genes directly bound by ZNF217 in B-ALL cells. Through CUT&RUN-seq, we identified genes that exhibited a significant reduction in ZNF217 occupancy upon *ZNF217* KD. These genes were termed ZNF217-bound targets (Figure 6D). We also conducted LSD1, H3K4me1, H3K4me2, and H3K27ac CUT&RUN-seq in KOPN-8 cells to evaluate the global effect of *ZNF217* KD on LSD1 binding and histone modifications. Based on these CUT&RUN-seq data, the ZNF217-bound targets were stratified into two subgroups: CoREST-dependent targets and CoREST-independent targets. Specifically, the ZNF217-bound target genes showing a marked decrease in LSD1 binding or increases in H3K4me1, H3K4me2, or H3K27ac levels following *ZNF217* KD, were categorized as being regulated by ZNF217 in a CoREST-dependent manner. Then, the remaining ZNF217-bound targets were classified as CoREST-independent targets (Figure 6D). In KOPN-8 cells, we identified 3,159 ZNF217-bound genes, with approximately two-thirds (2,142 genes) categorized as CoREST-independent targets (Figure 6E). Integrating analysis of RNA-seq with CUT&RUN-seq data revealed 61 ZNF217-bound target genes exhibiting more than two-fold upregulation following *ZNF217* depletion, with 49 of them classified as CoREST-independent targets (Figure 6F and Supplementary Table 4). Additionally, we identified 33 ZNF217-bound genes exhibiting more than two-fold downregulation following ZNF217 depletion, with 24 of them classified as CoREST-independent targets (Figure S7A and Supplementary Table 4). These findings suggest that, in B-ALL, ZNF217 regulates the expression of the majority (> 65%) of its downstream targets through a CoREST-independent mechanism.

We next conducted GSEA on these CoREST-independent targets. Our analysis revealed that the target genes that were CoREST-independent and upregulated upon *ZNF217* depletion were significantly enriched in pathways such as “TNFα signaling via NF-κB”, “UV response”, and “estrogen response” (Figure 6G). Amongst these core-enriched genes (Figure 6H), FOS (Fos proto-oncogene) has been reported to induce apoptotic cell death and/or cell-cycle arrest across various cancer types, including colon, prostate, nasopharyngeal cancer, and myeloid leukemia, as well as activating apoptosis in mouse lymphoid cells 74-79, but its role in B-ALL is still unclear. Our RNA-seq and CUT&RUN-seq analyses suggested that FOS is suppressed by ZNF217 in a CoREST-independent manner in KOPN-8 cells. Further qPCR results validated that *ZNF217* depletion significantly increased *FOS* mRNA levels in KOPN-8 cells (Figure 6I and 6J), suggesting that ZNF217 negatively regulates FOS expression in B-ALL.

**Figure 7:**
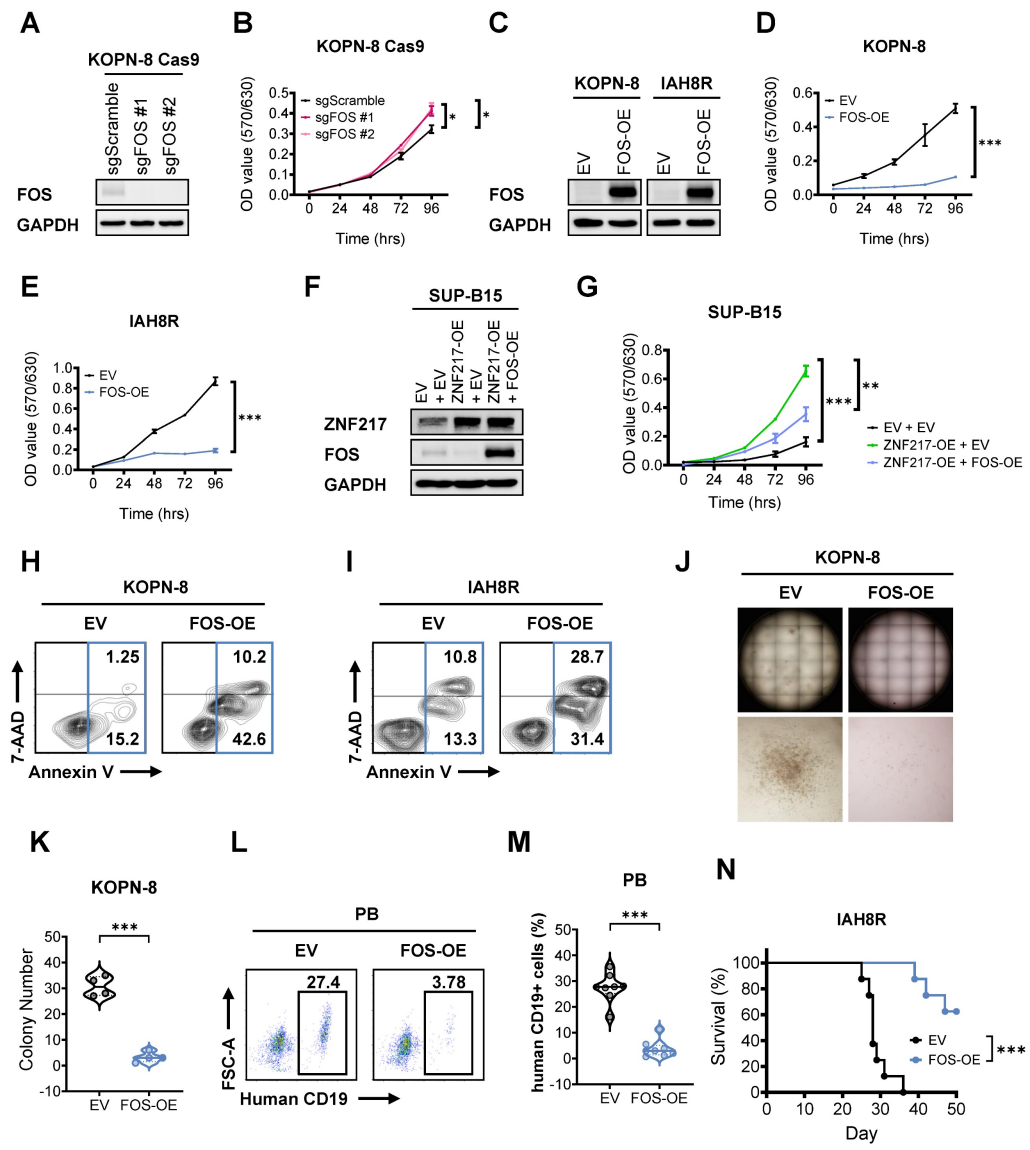
** FOS functions as a critical tumor suppressor downstream of ZNFF217 in B-ALL. (A)** *FOS* KO efficiency in KOPN-8 Cas9 cells as determined by western blotting. **(B)** Effect of *FOS* KO on the growth of KOPN-8 cells as determined by MTT assay. Data was presented as mean ± SD (n = 4 biological replicates). **(C)** *FOS* OE efficiency in KOPN-8 and IAH8R PDX cells as determined by western blotting. **(D)** Effect of *FOS* OE on the growth of KOPN-8 cells as determined by MTT assay. Data was presented as mean ± SD (n = 4 biological replicates). **(E)** Effect of *FOS* OE on the growth of IAH8R PDX cells as determined by MTT assay. Data was presented as mean ± SD (n = 4 biological replicates). **(F)** *ZNF217* OE efficiency and *FOS* OE efficiency in KOPN-8 cells, as determined by western blotting. **(G)** Effect of *ZNF217* OE on the growth of KOPN-8 cells and effect of *FOS* OE on the growth of *ZNF217*-overexpressing KOPN-8 cells, as determined by MTT assay. Data was presented as median with interquartile range (n = 4 biological replicates). **(H)** Effect of *FOS* OE on the apoptosis of KOPN-8 cells. **(I)** Effect of *FOS* OE on the apoptosis of IAH8R B-ALL PDX cells. **(J)** Effect of *FOS* OE on the colony-forming ability of KOPN-8 cells. **(K)** Quantification of the colony numbers in Figure 7J and Figure S8A. Data was presented as mean ± SD (n = 4 biological replicates). **(L)** Effect of *FOS* OE on leukemia burden in B-ALL PDX recipient (NSG) mice, as determined by flow cytometry measuring the percentage of human CD19⁺ cells in the peripheral blood of recipient mice. **(M)** Quantification of flow cytometry results presented in Figures 7M and S8B. Data was presented as median with interquartile range (n = 8 biological replicates). **(N)** Effect of *FOS* OE on overall survival in B-ALL PDX receipt mice. n = 8 for each group. The p values were calculated using two-tailed *t*-test (B, D, E, G, K, and M) and log-rank test (N). * p < 0.05; ** p < 0.01; *** p < 0.001.

To investigate the role of FOS in B-ALL, we performed both loss- and gain-of-function studies. Our MTT assays showed that *FOS* KO markedly promoted the growth of B-ALL cells (Figure 7A and 7B), and the opposite is true when it was overexpressed (Figure 7C-7E). These data indicated a tumor-suppressor role of FOS in B-ALL. Furthermore, overexpression of *FOS* in *ZNF217*-overexpressing KOPN-8 cells could partially reverse the growth advantage driven by *ZNF217* OE (Figure 7F and 7G), suggesting that FOS is a functionally important downstream target of ZNF217 in B-ALL cells. To further delineate FOS's specific function in B-ALL, we evaluated the effects of *FOS* OE in B-ALL cells. We found that *FOS* OE increased apoptosis in both KOPN-8 cell line and patient-derived IAH8R cells (Figures 7H and 7I). These findings are consistent with previous studies reporting FOS induces apoptosis in leukemia and lymphoid cells 79-81, and align with our observation that *ZNF217* depletion induces apoptosis in B-ALL cells (Figures 2G-2I and S2K-S2L), reinforcing the role of FOS as a tumor suppressor suppressed by ZNF217 in B-ALL. Moreover, *FOS* OE significantly impaired colony formation in KOPN-8 cells (Figures 7J-7K and S8A), underscoring its role in inhibiting B-ALL cell repopulation. To investigate FOS's function in B-ALL maintenance and progression *in vivo*, we transduced patient-derived IAH8R cells with either *FOS* or an empty vector and xenotransplanted them into NSG recipient mice. *FOS* OE significantly reduced leukemia burden in recipient mice (Figures 7L-7M and S8B) and markedly prolonged their overall survival (Figure 7N). These results highlight the significant tumor-suppressor role of FOS in suppressing B-ALL maintenance and progression *in vivo*. Taken together, our findings demonstrate that ZNF217 regulates the expression of its critical downstream targets through both CoREST-dependent and -independent mechanisms. Additionally, FOS acts as a critical tumor-suppressor and CoREST-independent target of ZNF217 in B-ALL.

## Discussion

In the present study, via a CRISPR-Cas9-based screen, we pinpointed *ZNF217* as one of the most essential genes for B-ALL cell survival/proliferation and further substantiated its oncogenic role through both *in vitro* and *in vivo* investigations. Our mechanistic study suggests that ZNF217's oncogenic function in B-ALL is partially mediated through its interaction with the CoREST complex, which influences histone modifications at ZNF217 target gene loci, thereby regulating the transcription/expression of ZNF217 target genes. While the interaction with the CoREST complex is important, our CUT&RUN-seq unveiled CoREST-independent mechanisms of ZNF217 in B-ALL. Of note, the majority of ZNF217 target genes in B-ALL were found to be CoREST-independent targets, underscoring the significance of the CoREST-independent pathway for ZNF217's oncogenic role in B-ALL.

We have also conducted a high-resolution CRISPR tiling screen and identified several functional essential regions within the ZNF217 protein, including the ZF1-4 zinc finger motifs essential for CoREST-binding and ZF6-7 for DNA-binding 51, 69, 72. Interestingly, our study revealed that the ZF5 zinc finger motif is also indispensable for maintaining the oncogenic function of ZNF217 in B-ALL. Nevertheless, the precise molecular mechanism of ZF5 remains undefined. Given our CUT&RUN-seq highlighting CoREST-independent mechanisms reliant on DNA binding, it is possible that the novel function associated with ZF5 might contribute a CoREST-independent but DNA-binding-dependent mechanism to the oncogenic role of ZNF217 in B-ALL. Nevertheless, systematical studies are warranted to test this possibility.

Notably, although previous studies reported that ZNF217 is implicated in m^6^A RNA methylation by either sequestering the m^6^A "writer" METTL3 or enhancing the transcription of the m^6^A "eraser" FTO 40-45, our findings suggest that ZNF217 is not associated with m^6^A modification in B-ALL. This identification indicates that the biological functions and molecular mechanisms of ZNF217 are highly context dependent.

Our RNA-seq analysis revealed that ZNF217 regulates the expression of genes involved in cell proliferation, survival, and apoptosis pathways (such as “TNFα signaling via NF-κB”, “E2F targets”, “G2M checkpoint”, and “apoptosis”). Notably, *FOS* is one of the top core-enriched genes in “TNFα signaling via NF-κB”, and ZNF217 significantly suppresses the expression of FOS in B-ALL via a CoREST-independent mechanism. Our *in vitro* and *in vivo* experiments have demonstrated that FOS suppresses B-ALL leukemogenesis and serves as a bona fide tumor-suppressor downstream target of ZNF217 in B-ALL. It is worth noting that FOS has been primarily associated with pro-oncogenic activity and poor overall survival 82, 83. However, several other studies appreciate the tumor-suppressor role of FOS in prostate cancer and rhabdomyosarcoma 84, 85. Moreover, FOS could induce apoptosis in hematopoietic cells 86. Thus, it would be very interesting to elucidate the mechanism that confers the oncogenic or tumor-suppressor role to FOS across different cancer types, though it is out of the scope of our current study. In our integrative bioinformatics analysis and pathway enrichment analysis, *FOS* stands out as one of the most significantly upregulated core-enriched genes in B-ALL following *ZNF217* depletion, but FOS is unlikely to be the sole critical downstream target of ZNF217 in B-ALL. Indeed, our analysis revealed additional candidate targets within the pathways. For instance, ZNF217 suppresses the expression of genes associated with immune responses, such as “TNFα signaling via NF-κΒ”, “interferon-γ response”, “inflammatory response”, and “interferon-α response”. Specifically, ZNF217 suppresses the expression of CD70, a cytokine that can induce cytotoxic T cell responses in B-ALL 87. This suggests ZNF217 might also play a role in evading immunosurveillance in B-ALL, underscoring the need for further research to comprehend ZNF217's impact within this context.

In summary, our research reveals ZNF217's pivotal oncogenic role in B-ALL. Our findings provide a foundation for the development of small molecule inhibitors targeting ZNF217, presenting a promising therapeutic approach for high-risk B-ALL treatment. This strategy holds potential for broader application across various cancers, including brain, breast, colon, gastric, liver, ovarian, pancreatic, and uterine cancers, in which *ZNF217* has been reported to be amplified and correlates with a worse prognosis 22-33. Our findings further reveal that ZNF217 regulates its essential target genes in B-ALL through both CoREST complex-dependent and -independent pathways. Additionally, our high-density ZNF217 CRISPR tiling screen identifies essential functional regions within ZNF217. Such data offers new perspectives on the molecular mechanisms underlying ZNF217's oncogenic functions, paving the way for developing targeted small-molecule inhibitors to suppress the ZNF217 signaling for the treatment of *ZNF217*-overexpressing cancers, such as high-risk B-ALL.

## Materials and Methods

### Cell lines and cell culture

KOPN-8 cells, sourced from the German Collection of Microorganisms and Cell Cultures (DSMZ), were cultivated in RPMI 1640 medium (11875119, Thermo Fisher Scientific) supplemented with 10% fetal bovine serum (FBS) (100-106, Gemini Bio-Products), 1% penicillin-streptomycin (15140122, Thermo Fisher Scientific), 10 mM HEPES (15630080, Thermo Fisher Scientific), and 2.5 μg/mL Plasmocin prophylactic (ant-mpp, InvivoGen). SUP-B15 cells, kindly provided by Dr. Markus Müschen, were cultured in RPMI 1640 medium supplemented with 20% FBS, 1% penicillin-streptomycin, 10 mM HEPES, and 2.5 μg/mL Plasmocin prophylactic. Patient-derived B-ALL cell lines—IAH8R, PDX2, and LAX7—were also generously provided by Dr. Markus Müschen. The IAH8R cells were derived from a relapsed B-ALL patient carrying the BCR-ABL1 fusion gene 88. The PDX2 cells originated from a diagnostic sample of a B-ALL patient also carrying a BCR-ABL1 fusion 89, 90. The LAX7 cells were established from a diagnostic sample of a BCR-ABL1-like B-ALL patient harboring an IL7R mutation 91-93. All patient-derived B-ALL cells were cultured in MEMα medium with nucleosides and GlutaMAX (32571036, Thermo Fisher Scientific) supplemented with 20% FBS, 1% penicillin-streptomycin, 10 mM HEPES, and 2.5 μg/mL Plasmocin prophylactic. HEK293T cells, obtained from the American Type Culture Collection (ATCC), were maintained in DMEM medium (10569010, Thermo Fisher Scientific) supplemented with 10% FBS, 1% penicillin-streptomycin, 10 mM HEPES, and 2.5 μg/mL Plasmocin prophylactic. All cell lines underwent authentication via Short Tandem Repeat (STR) analysis (Laragen, Inc.).

To generate KOPN-8 and SUP-B15 single clones stably expressing Cas9, the corresponding cells were transduced with lentiCas9-Blast (52962, Addgene) followed by antibiotic selection using 2-5 μg/mL blasticidin. Post-selection, these blasticidin-resistant cells were seeded into 96-well plates at a density of 0.5 cell per well to isolate single clones. The CRISPR editing efficiency of the single clones was assessed using an ipUSEPR-sgRFP reporter, adhering to an established protocol 94. Clones exhibiting high editing efficiency were reserved for subsequent studies.

To generate murine B-ALL cells transformed by BCR-ABL1, bone marrow cells were harvested from 6-8-week-old mice showing no inflammation signs. These cells were isolated by flushing the cavities of femurs and tibiae using RPMI 1640 medium containing 2% FBS and then passed through a 40 μm cell strainer. Erythrocytes within this mixture were eliminated using ammonium chloride solution (07850, STEMCELL Technologies). The remaining cells were then rinsed with PBS and cultured in IMDM medium with GlutaMAX (12440053, Thermo Fisher Scientific) supplemented with 20% FBS, 50 μM β-mercaptoethanol (M3148, Sigma-Aldrich), and 1% penicillin-streptomycin. For the selection of murine pre-B cells, the culture was further supplemented with 10 ng/mL recombinant mouse IL-7 (217-17, PeproTech) for 7 days. Subsequently, the murine pre-B cells were transduced with MSCV-BCR-ABL1-GFP retrovirus. After transduction, IL-7 was withdrawn from the culture to select the B-ALL cells transformed by BCR-ABL1.

To establish IAH8R and KOPN-8 cells that stably express firefly luciferase (termed IAH8R Luc and KOPN-8 Luc cells, respectively), these cell lines were transduced with pLenti-PGK-V5-Luc-Neo and subsequently selected with 1.0 mg/mL G418 Sulfate (10131027, Thermo Fisher Scientific). Post-selection, the luciferase efficacy in the cells was verified using the BioTek Synergy Neo2 Reader (Agilent Technologies).

All cell cultures were maintained at 37 °C in a 5% CO_2_ humidified incubator. Routine mycoplasma contamination checks were executed using the Mycoplasma PCR Detection Kit (G238, Applied Biological Materials), ensuring only uncontaminated cells were utilized in experiments.

### Lentiviral and retroviral transduction

Virus production was facilitated through the transfection of HEK-293T cells, using either the Effectene Transfection Reagent (301427, Qiagen) or the X-tremeGENE HP DNA Transfection Reagent (6366236001, Sigma-Aldrich), in accordance with manufacturer guidelines. Lentiviral particles were packaged with psPAX2 (Addgene) and pMD2.G (Addgene), while retroviral particles utilized pCL-ECO (Imgenex). Viral particles were harvested 48 h and 72 h post-transfection and then passed through a 0.45 μm syringe filter. In some instances, during the lentiviral preparation, the filtered supernatant underwent further concentration using the PEG-it Virus Precipitation Solution (LV810A-1, System Biosciences) and was stored at -80 °C until needed.

Viral transduction was conducted using either polybrene (H9268, Sigma-Aldrich) or RetroNectin (T100B, Takara). When using polybrene for transduction, cells were combined with the designated viruses and 4 μg/mL polybrene, followed by centrifugation at 32 °C at 1200 rpm for 2 h. In the RetroNectin method, viruses were loaded onto RetroNectin-coated, non-treated 6-well plates and centrifuged at 32 °C, 2000 × g for 2-5 h. Subsequently, cells were introduced to these plates and centrifuged at 32 °C, 600 × g for 30 min. 24 h post-transduction, the virus-containing medium was replaced with fresh culture medium. 48 h post-transduction, the transduced cells underwent antibiotic selection based on the specific antibiotic resistance conferred by the incorporated construct.

### RNA extraction, reverse transcription, and quantitative real-time PCR

For total RNA isolation, cells from each designated group were homogenized with QIAzol reagent (79306, QIAGEN). The homogenate was then mixed with chloroform and centrifuged at 13,000 × g at 4 °C for 15 min. From the upper aqueous phase, total RNA was precipitated using ethanol and further purified using the miRNeasy Kit (217004, QIAGEN). RNA concentration and purity were assessed by UV spectroscopy using a NanoDrop Spectrophotometer (Thermo Fisher Scientific). Subsequently, 500-1000 ng of total RNA underwent reverse transcription using the QuantiTect Reverse Transcription Kit (205314, QIAGEN) as per the manufacturer's protocol.

Quantitative real-time PCR (qPCR) was conducted in triplicate in a 384-well plate, with each reaction comprising 0.5 μL of 1:4 diluted cDNA, 1 μL of primer mix (5 μM each), 5 μL of Maxima SYBR Green qPCR Master Mix (FERK0253, Thermo Fisher Scientific), and 3.5 μL of nuclease-free water. The qPCR reactions were run on a QuantStudio 7 Flex PCR system (Thermo Fisher Scientific) under standard cycling conditions. Relative gene expression was determined using the comparative CT (ΔΔCT) method 95, with GAPDH or ACTB as internal controls. Primer details are provided in Supplementary Table 5.

### Western blotting

For protein extraction, cells were washed twice with PBS before being lysed on ice using RIPA buffer (R0278, Sigma-Aldrich) supplemented with 5 nM EDTA, 1 × Halt phosphatase inhibitor cocktail (78420, Thermo Fisher Scientific), and 1 × Halt protease inhibitor cocktail (78429, Thermo Fisher Scientific). Subsequently, cell extracts underwent centrifugation at 13,000 rpm for 20 min at 4 °C, and protein lysates in the supernatants were collected. The protein concentrations of these lysates were assessed using the Bio-Rad Protein Assay Dye Reagent Concentrate (5000006, Bio-Rad), referencing bovine serum albumin (5000007, Bio-Rad) as a standard. Lysates were then equilibrated in concentration using 4 × Laemmli Sample Buffer (1610747, Bio-Rad) supplemented with 10% β-mercaptoethanol (M3148, Sigma-Aldrich) and denatured at 95 °C for 10 min.

For Western blotting, lysates of equivalent protein amounts (20-40 μg each) were resolved on 10-15% SDS-PAGE gels and transferred onto 0.2 or 0.45 μm PVDF membranes. These membranes were then blocked using 5% non-fat milk (1706404XTU, Bio-Rad) in PBST and sequentially incubated with primary and secondary antibodies. Following this, chemiluminescent signals were detected using either the Pierce ECL Western Blotting Substrate (32106, Thermo Fisher Scientific) or the Amersham ECL Prime Western Blotting Detection Reagent (45010090, GE Healthcare). For histone modifications, Western blot signals were quantified using ImageJ software 96.

Primary antibodies utilized in the Western blotting included anti-ZNF217 (1:1000, ab124927, Abcam), anti-ZNF217 (1:1000, PA5-77093, Thermo Fisher Scientific), anti-CoREST (1:1000, 14567S, Cell Signaling Technology), anti-LSD1 (1:1000, 2139S, Cell Signaling Technology), anti-HDAC1 (1:1000, sc-7872, Santa Cruz Biotechnology), anti-HDAC2 (1:1000, 67165-1-Ig, Proteintech), anti-H3K27ac (1:1000, 39034, Active Motif), anti-H3K4me1 (1:2000, 91290, Active Motif), anti-H3K4me2 (1:2000, 91322, Active Motif), anti-YTHDF2 (1:1000, 24744-1-AP), anti-METTL3 (1:1000, ab195352, Abcam), anti-METTL14 (1:1000, HPA038002, Sigma-Aldrich), anti-METTL16 (1:1000, HPA020352, Sigma-Aldrich), anti-IGF2BP1 (1:1000, 8482S, Cell Signaling Technology), anti-IGF2BP2 (1:1000, 14672S, Cell Signaling Technology), anti-PCIF1 (1:1000, ab205016, Abcam), anti-FOS (1:1000, 2250T, Cell Signaling Technology), anti-GAPDH (1:1000, sc-47724, Santa Cruz Biotechnology), and anti-β-Actin (1:5000, 3700, Cell Signaling Technology). Secondary antibodies included Goat Anti-Mouse IgG H&L (HRP) (ab6789, Abcam) and Goat Anti-Rabbit IgG H&L (HRP) (ab6721, Abcam).

### sgRNA library design and preparation

The sgRNA library for the DNA/RNA methylation gene panel screen, previously described 97, includes 900 sgRNAs targeting 36 genes associated with RNA and DNA methylation machinery (25 sgRNAs per gene), 22 sgRNAs for common essential genes like *MYC* and *BRD4*, and 41 sgRNAs for non-essential sequences (Supplementary Table 1). For the ZNF217-tiling screen, the library contains 416 sgRNAs targeting ZNF217's coding regions at a density of 7.5 bp per sgRNA, 22 sgRNAs for common essential genes, and 40 sgRNAs for non-essential sequences (Supplementary Table 1).

The sgRNA sequences were designed using the Genetic Perturbation Platform (Broad Institute) 70. The corresponding oligonucleotides were synthesized via a microarray (CustomArray). Subsequently, the oligonucleotides were cloned into the ipUSEPR lentiviral sgRNA vector, which facilitates hU6-driven expression of the designated sgRNA and EF-1α-driven expression of both a puromycin-resistance gene and an RFP reporter, using the BsmBI restriction sites. Following molecular cloning, the resultant plasmids underwent validation by Sanger sequencing.

### CRISPR library screen

CRISPR library screens were carried out in triplicate, adapting a previously described protocol with minor adjustments 94. Briefly, lentiviruses of the sgRNA library were prepared and pre-titrated to achieve an infection rate of 10-15%, as determined by RFP expression via flow cytometry, in B-ALL (KOPN-8 or SUP-B15) Cas9 single clone cells. Following this, the cells were transduced with the pre-determined quantity of lentiviruses. 48 h post-transduction, the infection efficiency was verified using flow cytometry, after which 2 μg/mL of puromycin was added for selection. At 72 h after transduction, half of the cells were harvested to mark the "initial point" of the screen. The remaining cells were cultivated with puromycin selection at 37 °C in a 5% CO_2_ humidified incubator. Once the accumulated cell amplification reached a range of 5000 × to 10000 ×, they were harvested, marking the "final point" of the screen. Throughout the lentiviral transduction, cultivation, and sample collection processes, cell numbers were meticulously calculated to ensure a minimum of 1000 × coverage of the sgRNA library.

From cells collected at both the "initial" and "final" points, genomic DNA was extracted. Thereafter, the integrated sgRNAs in these samples underwent PCR amplification using NEBNext Ultra II Q5 (M0544L, NEB), employing the primers DCF01 (5'-CTTGTGGAAAGGACGAAACACCG-3') and DCR03 (5'-CCTAGGAACAGCGGTTTAAAAAAGC-3'). Following PCR, the samples underwent high-throughput sequencing using the NextSeq 550 System (Illumina).

### CRISPR screen data analysis

To quantify sgRNA read counts, the 20-nucleotide sequences that matched the sgRNA backbone structure (5-CACCG and GTTT-3') were extracted and mapped to sgRNA library sequences using Bowtie2 98. The frequency of each sgRNA was calculated as the ratio of its read counts to the total read counts of the library.

In the DNA/RNA methylation gene panel screen, candidate genes under negative selection were ranked by robust rank aggregation (RRA) scores, using the Model-based Analysis of Genome-wide CRISPR-Cas9 Knockout (MAGeCK) algorithm 99. The CRISPR score for each sgRNA was calculated as the log10-fold change in its frequency from "initial" to "final" points, normalized against the average log10-fold change of sgRNAs targeting non-essential sequences (set at 0.0).

In the ZNF217-tiling screen, CRISPR scores were calculated similarly but normalized against the median scores of negative control sgRNAs targeting non-essential sequences (set at 0.0) and positive control sgRNAs targeting common essential genes (set at -1.0). For 2D annotation, the CRISPR scores underwent Gaussian kernel smoothing in R, with the average score computed for each peptide position over trinucleotide codons.

### Growth competition assay

In growth competition assays, the following sgRNAs were utilized: 8 *ZNF217* sgRNAs selected from the 25 initially used in CRISPR screening, 2 positive control sgRNAs targeting *BRD4* and *MYC* respectively, and 2 negative control sgRNAs targeting firefly and Renilla luciferase genes respectively. These sgRNAs were individually cloned into the ipUSEPR lentiviral backbone, which contains an RFP reporter.

Following lentiviral preparation, KOPN-8 Cas9 and SUP-B15 Cas9 single clone cells were transduced with each distinct sgRNA in 96-well plates. Lentiviruses for each sgRNA were added to individual wells using a serial dilution strategy, aiming for infection efficiency approximate to 50%. Specifically, 1 × 10^4^ KOPN-8 Cas9 cells were used for each infection reaction, while 2 × 10^4^ SUP-B15 Cas9 cells were used for their respective reactions. Infections were conducted in triplicate, with each replicate including some uninfected cells serving as negative controls for measuring the percentage of RFP-positive cells.

Two days after infection, half of the cells from each reaction were taken for infection efficiency analysis using the Attune flow cytometer. The infection efficiency was determined by the proportion of RFP-positive cells. For each sgRNA, reactions exhibiting approximately 50% infection efficiency were selected for further study.

Following this, cells from the selected reactions were further cultivated and monitored in 96-well plates for 28 days. To combat evaporation, PBS was added into the inter-well spaces. Every 4 days, half of the cells from each well were analyzed for the percentage of RFP-positive cells using the Attune flow cytometer, while the remaining cells continued cultivation.

### Cell proliferation assay

Cell proliferation and growth were assessed using the MTT-based CellTiter 96-Non-Radioactive Cell Proliferation Assay (G4000, Promega) as per the manufacturer's guidelines. In brief, cells were seeded in quadruplicate into flat-bottomed, non-treated 96-well plates. Each well contained 5,000 to 25,000 viable cells in 100 μL of full cell culture medium. At designated time points, 15 μL of dye solution was introduced to each well. Following an incubation period of 4-5 h at 37 °C in a 5% CO_2_ humidified incubator, 100 μL of solubilization/stop solution was added to quench the reaction. Absorbance readings were taken the subsequent day at 570 nm (and a reference at 630 nm) using BioTek Synergy Neo2 Reader (Agilent Technologies).

### Apoptosis assay

Cell apoptosis was determined using the PE Annexin V Apoptosis Detection Kit (559763, BD Biosciences) in accordance with the manufacturer's guidelines. In brief, cells underwent two washes with cold PBS and were subsequently stained with PE-labeled annexin V and 7-AAD. This staining was conducted at room temperature, shielded from light, for 15 min. Following staining, the cells were immediately analyzed via flow cytometry on an LSRFortessa X-20 Cell Analyzer (BD Biosciences). The resultant data were interpreted using FlowJo software (FlowJo, LLC).

### Colony-forming assay

In the colony-forming assay using BCR-ABL1 transformed mouse pre-B cells, the cells were transduced with the specified shRNAs and selected using 1 μg/mL puromycin. After selection, 1 × 10⁵ cells were seeded in 1.5 mL of murine MethoCult medium M3231 (StemCell Technologies) supplemented with 1 μg/mL puromycin. The cells were cultured in 35 mm dishes at 37 °C in a humidified incubator with 5% CO_2_. Each condition was performed with two technical replicates. After 21 days, colonies were manually counted.

In the colony-forming assay using KOPN-8 cells, cells were transduced with either *FOS* or an empty vector and selected using 10 μg/mL blasticidin. Subsequently, 1 × 10³ cells were seeded in 0.5 mL of Human Base Colony Gel (1101, ReachBio) supplemented with 10 ng/mL recombinant human IL-7 (207-IL-010, R&D Systems) and 10 μg/mL blasticidin. The cells were cultured in a 24-well plate at 37 °C in a humidified incubator with 5% CO_2_. Each condition was conducted with four technical replicates. After 12 days, images were captured using an Olympus IX83 microscope (Olympus Corporation), and colonies were manually counted.

### Animals

NOD.Cg-*Prkdc^scid^ Il2rg^tm1Wjl^*/SzJ (NSG) mice, originally obtained from the Jackson Laboratory, were bred and housed under pathogen-free conditions in the certified animal facility at City of Hope. The mice were maintained under a 12 h light/12 h dark cycle and provided with *ad libitum* access to food and water. All mouse breeding and experimental procedures were conducted in accordance with research protocols approved by the Institutional Animal Care and Use Committee (IACUC) at City of Hope.

### B-ALL PDX and xenograft models

NSG mice aged 6-8 weeks, free from signs of inflammation, were used as recipients for xenotransplantation in our B-ALL PDX and xenograft models. Each experimental group consisted of 8 mice, including both males and females, randomly allocated to different experimental conditions.

To examine the effects of *ZNF217* KD, IAH8R and KOPN-8 cells stably expressing firefly luciferase (designated as IAH8R Luc and KOPN-8 Luc, respectively) were transduced with *ZNF217* shRNAs or a scramble control shRNA. Following lentiviral transduction, the cells underwent puromycin selection, and knockdown efficiency was validated via Western blotting. Four to five days post-transduction, recipient mice received semi-lethal irradiation at 2.5 Gy. Subsequently, 0.2 × 10^6^ IAH8R Luc cells or 0.1 × 10^6^ KOPN-8 Luc cells, transduced with the designated shRNA, were resuspended in 100 µL PBS and intravenously injected into the irradiated mice. B-ALL progression was monitored using D-luciferin-induced bioluminescence imaging. Mice displaying terminal illness symptoms such as weight loss, labored breathing, hunched posture, reduced mobility, and paralysis were humanely euthanized via CO_2_ inhalation. Spleen-to-body weight ratios were analyzed post-euthanasia to evaluate disease impact.

To examine the effects of *FOS* OE, IAH8R cells were transduced with either *FOS* or an empty vector. After lentiviral transduction and blasticidin selection, the overexpression efficiency was validated via Western blotting. Subsequently, 2 × 10⁶ cells were resuspended in 100 µL of PBS and intravenously injected into NSG recipient mice. After 21 days, peripheral blood was collected from each mouse to assess leukemia burden by measuring the percentage of human CD19⁺ cells. Mouse survival was monitored, and mice exhibiting terminal illness symptoms were humanely euthanized using CO_2_ inhalation.

### *In vivo* bioluminescence imaging

In the recipient mice, bioluminescent signals were generated through the oxidation of D-luciferin, a process catalyzed by firefly luciferase, which had been stably transduced into the injected B-ALL cells. The detection of bioluminescence followed a protocol adapted from previous studies 100. Briefly, 100 µL of a 25 mg/mL D-luciferin in PBS solution was injected intraperitoneally to the mice. Immediately post-injection, the mice were anesthetized using isoflurane inhalation. Imaging took place 10 min post-injection to capture the bioluminescent signals, utilizing the Lago X In Vivo Imaging System (Spectral Instruments Imaging). The bioluminescent signals were subsequently visualized and quantified using the Aura Imaging Software (Spectral Instruments Imaging).

### Evaluation of leukemia burden by flow cytometry

The effect of *FOS* OE on leukemia burden in B-ALL PDX recipient (NSG) mice was assessed by measuring the percentage of human CD19⁺ cells in the peripheral blood. Briefly, 30-40 μL of peripheral blood was collected from the tail vein of each recipient mouse 21 days after transplantation. The red blood cells were lysed using 5 mL of Ammonium Chloride Solution (07850, STEMCELL Technologies) for 10 min at 4 °C. The remaining nucleated cells were washed twice with cold PBS and incubated with 2.5 μg of Human BD Fc Block (564219, BD Biosciences) in 100 μL of eBioscience™ Flow Cytometry Staining Buffer (00-4222-26, Thermo Fisher Scientific) for 10 min at room temperature. Subsequently, the cells were incubated with 20 μL of PE Mouse Anti-Human CD19 antibody (555413, BD Biosciences) in the dark for 30 min at 4 °C. After incubation, the cells were washed once with cold PBS and resuspended in 300 μL of FACS buffer (2% FBS in PBS) supplemented with 1 μg/mL DAPI. Flow cytometry analysis was performed using an LSRFortessa X-20 Cell Analyzer (BD Biosciences), and data were interpreted using FlowJo software (FlowJo, LLC).

### Co-immunoprecipitation (co-IP)

To prepare B-ALL cell lysates, 2-5 × 10^7^ cells were washed twice with cold PBS and then lysed on ice for 30 min using 1.5 mL RIPA buffer (R0278, Sigma-Aldrich) supplemented with 5 nM EDTA, 1 × Halt phosphatase inhibitor cocktail (78420, Thermo Fisher Scientific), and 1 × Halt protease inhibitor cocktail (78429, Thermo Fisher Scientific). The mixture was subsequently centrifuged at 4 °C at 13,000 g for 20 min, and the supernatant was collected as the cell lysate for co-IP.

The cell lysate was incubated with 50 μL Protein A/G magnetic beads (88803, Thermo Fisher Scientific) and rotated gently at 4 °C for 1 h to minimize non-specific binding. After removing the beads using a magnetic stand, the pre-cleared lysate was evenly distributed among designated groups (500-1000 μg each), reserving a 1% aliquot as an input control. These lysates were then incubated with their respective antibodies at 4 °C with gentle rotation for an hour. Afterwards, 25 μL of Protein A/G magnetic beads were added to each lysate-antibody mixture, followed by overnight incubation at 4 °C with gentle rotation. Post-incubation, the antibody-protein complexes bound to the Protein A/G magnetic beads were captured using a magnetic stand and washed three times with IP washing buffer. Each co-IP sample was then eluted in 50 μL 2 × Laemmli buffer (1610737, Bio-Rad) without β-mercaptoethanol by incubation at 50 °C for 10 min. Subsequently, each eluate was mixed with 50 μL RIPA buffer containing 20% β-mercaptoethanol (M3148, Sigma-Aldrich). The 1% input control was diluted to 100 μL in 1 × Laemmli buffer with 10% β-mercaptoethanol. All co-IP and input samples were denatured at 95 °C for 10 min before being subjected to Western blot analysis.

The antibodies employed in the co-IP assays included anti-Flag (F3165, Sigma-Aldrich), anti-ZNF217 (A303-265A, Thermo Fisher Scientific), anti-LSD1 (2139S, Cell Signaling Technology), anti-mouse IgG (12-371, Sigma-Aldrich), and anti-rabbit IgG (NI01, Sigma-Aldrich). The IP washing buffer comprised 10 mM Tris-HCl (pH 7.5), 1 mM EDTA, 150 mM NaCl, 1% Triton X-100, and 0.2 mM sodium orthovanadate.

### m^6^A dot blot

The m^6^A dot blot assays were conducted using either total RNA or poly(A)^+^ mRNA. Total RNA was purified using the miRNeasy Mini Kit (217004, QIAGEN), and poly(A)^+^ mRNA was isolated using the PolyATtract mRNA Isolation Systems (Z5310, Promega), both according to their respective manufacturer's instructions.

In the dot blot assays, total RNA or poly(A)^+^ mRNA was first denatured in RNA incubation buffer (comprising 65.7% formamide, 7.77% formaldehyde, and 1.33 × MOPS) at 65 °C for 5 min, followed by immediate chilling on ice. Each sample was then mixed with an equal volume of 20 × standard saline citrate (SSC) buffer. The specified amounts of RNA were subsequently loaded onto a Hybond-N^+^ hybridization membrane (RPN303B, GE Healthcare) positioned in a Bio-Dot Apparatus (170-6545, Bio-Rad). Following this, the membrane was air-dried at room temperature, followed by crosslinking under 254 nm UV light for 5 min. To verify equal RNA loading, the membrane was stained with 0.02% methylene blue in 0.3 M sodium acetate (pH 5.2). After imaging, the membrane was blocked with 5% non-fat milk in 1 × PBST for 1 h, followed by an overnight incubation at 4 °C with an anti-m^6^A antibody (1:5000, 202003, Synaptic Systems). The next day, after three washes in 1 × PBST, the membrane was incubated with an HRP-conjugated secondary antibody (1:5000, ab6789, Abcam) at room temperature for 1 h. Chemiluminescent signals were detected using Pierce ECL Western Blotting Substrate (32106, Thermo Fisher Scientific) or Amersham ECL Prime Western Blotting Detection Reagent (45010090, GE Healthcare).

### UHPLC-QQQ-MS/MS

Ultra-high pressure liquid chromatography coupled with triple-quadrupole tandem mass spectrometry (UHPLC-QQQ-MS/MS) was utilized to measure N6-methyladenosine (m^6^A) levels in either total RNA or poly(A)^+^ mRNA. Total RNA was purified using the miRNeasy Mini Kit (217004, QIAGEN), and poly(A)^+^ mRNA was isolated from total RNA by two rounds of purification using Dynabeads Oligo(dT)_25_ (61005, Invitrogen), following their respective manufacturer's protocols.

For nucleoside preparation, the RNA samples underwent a two-step digestion process 101. Initially, 100 ng of RNA was digested with 0.5 U Nuclease P1 (N8630, Sigma-Aldrich) in 10 μL of 20 mM NH4OAc (pH 5.5) at 42 °C for 2 h. This was followed by digestion with 0.5 U FastAP Thermosensitive Alkaline Phosphatase (EF0651, Thermo Scientific) at 37 °C for another 2 h. Post-digestion, samples were diluted in LC-MS grade water containing 0.2 fmol/μL m^6^A-d3 as an internal standard. Subsequently, samples were purified by heating at 65 °C for 10 min and centrifuging at 14,000 rpm for 10 min. The resulting supernatants were used for quantification.

UHPLC-QQQ-MS/MS analysis was conducted on an Agilent 6410 Triple Quadrupole Mass Spectrometer paired with an Agilent 1290 Infinity LC II System (Agilent Technologies). Nucleosides were separated on a C18 column (00A-4475-AN, Phenomenex). Nucleoside detection was based on retention time and mass-to-charge ratio (m/z) transitions, specifically monitoring m/z transitions of 268-to-136 for adenine and 282.1-to-150.1 for m^6^A. Nucleoside amounts were calibrated against standard curves derived from the internal standard. The m^6^A level in each RNA sample was then calculated as the m^6^A-to-A ratio.

### Conservation analysis

Conservation analysis of ZNF217 protein sequences was conducted in Python, employing a Jensen-Shannon divergence-based method and the BLOSUM62 substitution matrix 71. The analysis utilized NCBI reference sequences for ZNF217 proteins from 8 species, including *Homo sapiens* (NP_006517.1), *Pan troglodytes* (XP_009435702.1), *Macaca mulatta* (XP_015004258.1), *Equus caballus* (XP_023482537.1), *Sus scrofa* (NP_001116689.1), *Felis catus* (XP_023106855.2), *Cricetulus griseus* (XP_007651300.1), and *Mus musculus* (NP_001028471.1). Conservation scores for deletion sites within the human ZNF217 protein were omitted prior to creating a heatmap that maps these scores to the human ZNF217 protein sequence.

### RNA sequencing and data analysis

For RNA sequencing, single-cloned KOPN-8 Cas9 cells transduced with either *ZNF217* sgRNA (sgZNF217 #1 or sgZNF217 #2) or a scrambled sgRNA control were used. Following validation of *ZNF217* knockout efficiency, cells were harvested to isolate total RNA using QIAzol reagent (79306, QIAGEN) and the miRNeasy Kit (217004, QIAGEN) according to the manufacturer's instructions. RNA quality was verified through agarose gel electrophoresis and Bioanalyzer assays (2100 Bioanalyzer system, Agilent), evaluating RNA size, integrity, purity, and concentration.

The double-stranded cDNA libraries were constructed by Novogene Corporation Inc. (Sacramento, CA). In brief, mRNA was enriched from the RNA samples using oligo(dT) beads, then underwent random fragmentation, reverse transcription, second-strand synthesis, and sequencing adaptor ligation. The adaptors used were 5'-AGATCGGAAGAGCGTCGTGTAGGGAAAGAGTGT-3' (5' Adapter) and 5'-GATCGGAAGAGCACACGTCTGAACTCCAGTCAC-3' (3' Adapter). Libraries were quantified using a Qubit 2.0 fluorometer (Thermo Fisher Scientific) and assessed for size distribution and effective concentration using the Agilent 2100 Bioanalyzer and RT-qPCR, respectively. Sequencing was performed on a NovaSeq 6000 System (Illumina) with 150 bp paired-end reads. Quality of the raw sequencing data was checked by Phred quality scores 102, and reads containing adaptors, undetermined bases, or low-quality bases were removed prior to analysis.

Sequencing reads were mapped to the human GRCh38 reference genome sequences using STAR 103. The expression of genes, including read counts and transcript per million (TPM), were calculated using RSEM 104. DESeq2 was used to assess dispersion and fold change in gene expression following ZNF217 knockout 105. Gene Set Enrichment Analysis (GSEA) was conducted using clusterProfiler4, referencing hallmark gene sets from the Molecular Signatures Database (MSigDB) 106, 107.

### CUT&RUN sequencing and data analysis

CUT&RUN assays were conducted on KOPN-8 cells transduced with either a *ZNF217*-targeting shRNA (shZNF217 #1) or a scrambled shRNA control using the CUTANA ChIC/CUT&RUN Kit (14-1048, EpiCypher), following the manufacturer's instructions. In the ZNF217 and LSD1 groups, live cells were used, while in the H3K4me1, H3K4me2, and H3K27ac groups, cells were lightly cross-linked with 0.1% formaldehyde for 1 min. The antibodies employed included anti-ZNF217 (A303-265A, Thermo Fisher Scientific), anti-LSD1 (2139S, Cell Signaling Technology), anti-H3K4me1 (5326T, Active Motif), anti-H3K4me2 (9725T, Active Motif), and anti-H3K27ac (39034, Active Motif).

For each sample, 10 ng of CUT&RUN-enriched DNA was used for library construction, employing the CUTANA CUT&RUN Library Prep Kit (14-1002, EpiCypher). Index primers used included CUTANA_i501, CUTANA_i502, CUTANA_i503, CUTANA_i504, CUTANA_i505, CUTANA_i506, CUTANA_i707, and CUTANA_i708. Library quality was assessed using an Agilent Bioanalyzer, and sequencing was performed on a NovaSeq System (Illumina) with 100 bp paired-end reads.

Raw sequencing data was quality-checked by FastQC, followed by alignment to the human genome (hg38) using Bowtie2 108. Enriched peaks were identified using MACS2 in narrow peak mode, with peaks having an FDR ≤ 0.05 considered significantly enriched 109. Peak annotation considered promoters as regions from -1 kb to +100 bp relative to the transcription start site 110. Enhancer annotations were sourced from the ENCODE database and super-enhancer annotations from the Sedb database 111, 112.

## Supplementary Material

Supplementary figures.

Supplementary tables.
