# Supplementary material for: CRISPR screening reveals ZNF217 as a vulnerability in high-risk B-cell acute lymphoblastic leukemia
Source: Theranostics. 2025 Feb 18;15(8):3234–56. doi: 10.7150/thno.100295 (PMC11905140; doi:10.7150/thno.100295)
Supplement: Supplementary file 1 — Supplementary figures. [file thnov15p3234s1.pdf]

Figure S1

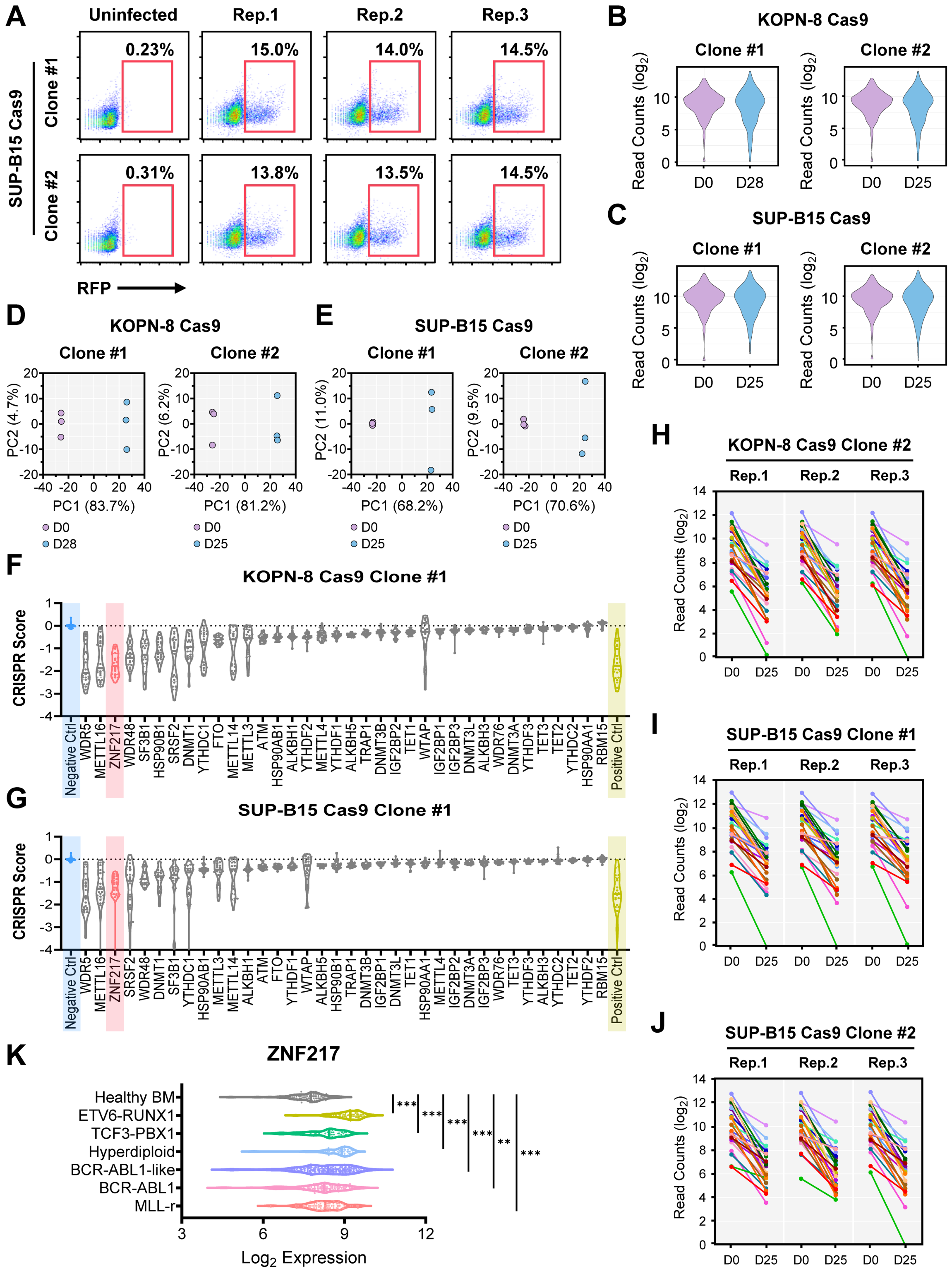
A
Uninfected
Rep.1
Rep.2
Rep.3
SUP-B15 Cas9
Clone #1
Clone #2
0.23%
15.0%
14.0%
14.5%
0.31%
13.8%
13.5%
14.5%
RFP
B
KOPN-8 Cas9
C
SUP-B15 Cas9
Clone #1
Clone #2
Read Counts (log2)
D0
D28
D25
D
KOPN-8 Cas9
E
SUP-B15 Cas9
PC1 (83.7%)
PC2 (4.7%)
PC1 (81.2%)
PC2 (6.2%)
PC1 (68.2%)
PC2 (11.0%)
PC1 (70.6%)
PC2 (9.5%)
F
KOPN-8 Cas9 Clone #1
G
SUP-B15 Cas9 Clone #1
CRISPR Score
Negative Ctrl
Positive Ctrl
H
KOPN-8 Cas9 Clone #2
I
SUP-B15 Cas9 Clone #1
J
SUP-B15 Cas9 Clone #2
K
ZNF217
Healthy BM
ETV6-RUNX1
TCF3-PBX1
Hyperdiploid
BCR-ABL1-like
BCR-ABL1
MLL-r
Log2 Expression

Figure S1. CRISPR screen identifies essential genes for the survival of B-ALL cells. (Related to Figure 1)

(A) The lentiviral transduction efficiency of sgRNA library in CRISPR screen using SUP-B15 Cas9 single clones.
(B) The distribution of sgRNA read counts in the NGS samples collected at the "initial" and "final" points of CRISPR screen using KOPN-8 Cas9 single clones.
(C) The distribution of sgRNA read counts in the NGS samples collected at the "initial" and "final" points of CRISPR screen using SUP-B15 Cas9 single clones.
(D) PCA analysis of NGS samples collected at the "initial" and "final" points of CRISPR screen using KOPN-8 Cas9 single clones.
(E) PCA analysis of NGS samples collected at the "initial" and "final" points of CRISPR screen using SUP-B15 Cas9 single clones.
(F) The CRISPR scores of sgRNAs targeting the 36 RNA and DNA methylation machinery-associated genes in CRISPR screen using KOPN-8 Cas9 single clones. The CRISPR scores have been normalized against the mean score of the negative control sgRNAs (set at 0.0).
(G) The CRISPR scores of sgRNAs targeting the 36 RNA and DNA methylation machinery-associated genes in CRISPR screen using SUP-B15 Cas9 single clones. The CRISPR scores have been normalized against the mean score of the negative control sgRNAs (set at 0.0).
(H) Read counts of the 25 sgRNAs targeting *ZNF217* in KOPN-8 Cas9 Clone #2.
(I) Read counts of the 25 sgRNAs targeting *ZNF217* in SUP-B15 Cas9 Clone #1.
(J) Read counts of the 25 sgRNAs targeting *ZNF217* in SUP-B15 Cas9 Clone #2.
(K) Expression of ZNF217 across different B-ALL subtypes and in healthy bone marrow. ZNF217 expression data were sourced from the Microarray Innovations in Leukemia (MILE) study (accession GSE13159). n = 70 for MLL-r; n = 122 for BCR-ABL1; n = 237 for BCR-ABL1-like; n = 40 for hyperdiploid; n = 36 for TCF3-PBX1; n = 58 for ETV6-RUNX1; n = 73 for healthy BM.
The p values were calculated using a two-tailed *t*-test. ** $p < 0.01$; *** $p < 0.001$.

Figure S2

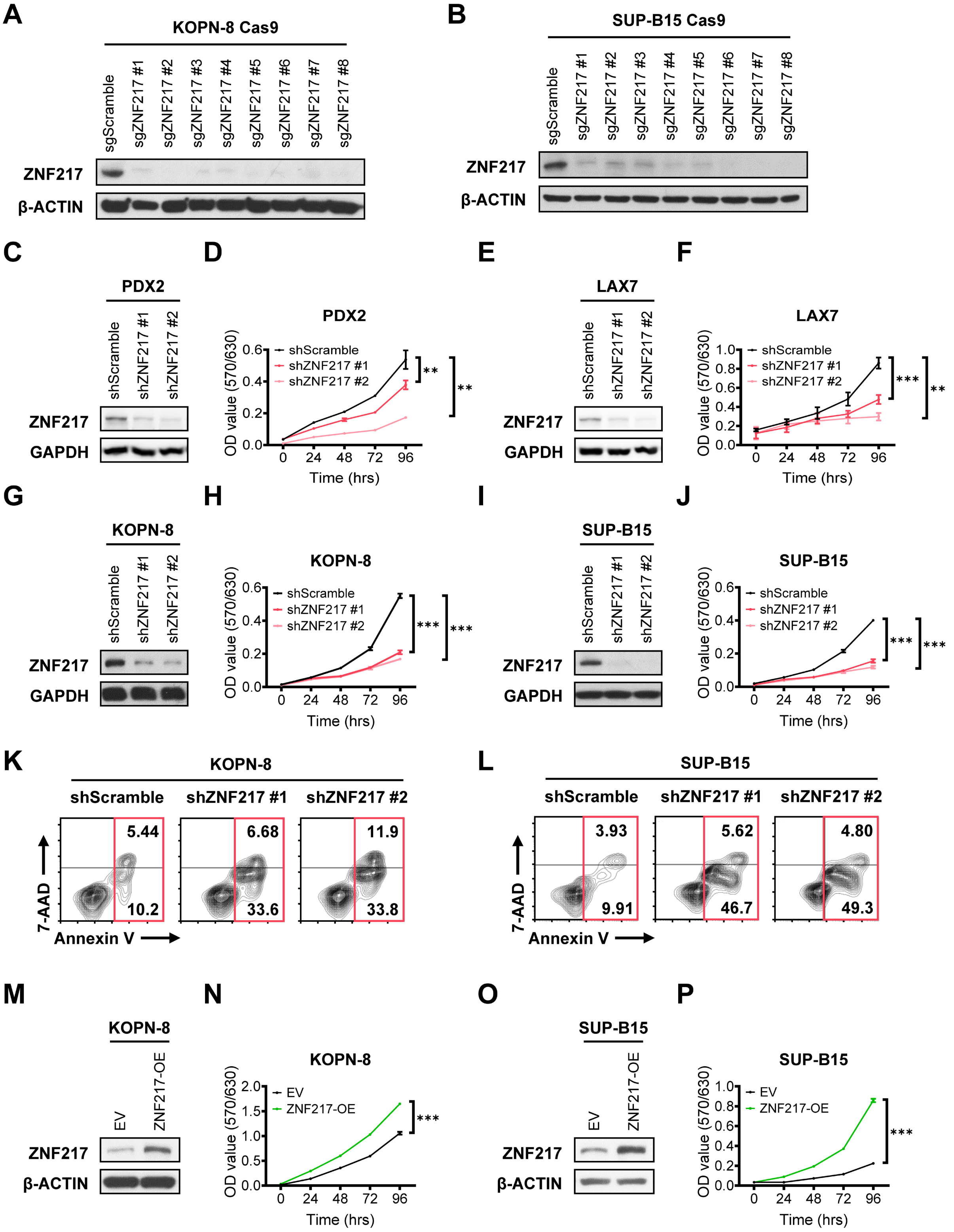

A
KOPN-8 Cas9
sgScramble
sgZNF217 #1
sgZNF217 #2
sgZNF217 #3
sgZNF217 #4
sgZNF217 #5
sgZNF217 #6
sgZNF217 #7
sgZNF217 #8
ZNF217
β-ACTIN
B
SUP-B15 Cas9
sgScramble
sgZNF217 #1
sgZNF217 #2
sgZNF217 #3
sgZNF217 #4
sgZNF217 #5
sgZNF217 #6
sgZNF217 #7
sgZNF217 #8
ZNF217
β-ACTIN
C
PDX2
shScramble
shZNF217 #1
shZNF217 #2
ZNF217
GAPDH
D
PDX2
shScramble
shZNF217 #1
shZNF217 #2
OD value (570/630)
Time (hrs)
**
**
E
LAX7
shScramble
shZNF217 #1
shZNF217 #2
ZNF217
GAPDH
F
LAX7
shScramble
shZNF217 #1
shZNF217 #2
OD value (570/630)
Time (hrs)
***
**
G
KOPN-8
shScramble
shZNF217 #1
shZNF217 #2
ZNF217
GAPDH
H
KOPN-8
shScramble
shZNF217 #1
shZNF217 #2
OD value (570/630)
Time (hrs)
***
***
I
SUP-B15
shScramble
shZNF217 #1
shZNF217 #2
ZNF217
GAPDH
J
SUP-B15
shScramble
shZNF217 #1
shZNF217 #2
OD value (570/630)
Time (hrs)
***
***
K
KOPN-8
shScramble
shZNF217 #1
shZNF217 #2
5.44
10.2
6.68
33.6
11.9
33.8
7-AAD
Annexin V
L
SUP-B15
shScramble
shZNF217 #1
shZNF217 #2
3.93
9.91
5.62
46.7
4.80
49.3
7-AAD
Annexin V
M
KOPN-8
EV
ZNF217-OE
ZNF217
β-ACTIN
N
KOPN-8
EV
ZNF217-OE
OD value (570/630)
Time (hrs)
***
O
SUP-B15
EV
ZNF217-OE
ZNF217
β-ACTIN
P
SUP-B15
EV
ZNF217-OE
OD value (570/630)
Time (hrs)
***

Figure S2. ***ZNF217*** **KO efficacy in B-ALL cells, and effects of** ***ZNF217*** **KD and OE on B-ALL cell growth and/or apoptosis. (Related to Figure 2)**

(A) KO efficacy of the 8 *ZNF217*-targeting sgRNAs, which were used in the growth competition assay, in KOPN-8 Cas9 cells.
(B) KO efficacy of the 8 *ZNF217*-targeting sgRNAs, which were used in the growth competition assay, in SUP-B15 Cas9 cells.
(C) *ZNF217* KD efficacy in PDX2 B-ALL PDX cells.
(D) Effect of *ZNF217* KD on the growth of PDX2 B-ALL PDX cells as determined by MTT assay. Data was presented as mean ± SD (n = 4 biological replicates).
(E) *ZNF217* KD efficacy in LAX7 B-ALL PDX cells.
(F) Effect of *ZNF217* KD on the growth of LAX7 B-ALL PDX cells as determined by MTT assay. Data was presented as mean ± SD (n = 4 biological replicates).
(G) *ZNF217* KD efficacy in KOPN-8 B-ALL cells.
(H) Effect of *ZNF217* KD on the growth of KOPN-8 B-ALL cells as determined by MTT assay. Data was presented as mean ± SD (n = 4 biological replicates).
(I) *ZNF217* KD efficacy in SUP-B15 B-ALL cells.
(J) Effect of *ZNF217* KD on the growth of SUP-B15 B-ALL cells as determined by MTT assay. Data was presented as mean ± SD (n = 4 biological replicates).
(K) Effect of *ZNF217* KD on the apoptosis of KOPN-8 B-ALL cells.
(L) Effect of *ZNF217* KD on the apoptosis of SUP-B15 B-ALL cells.
(M) *ZNF217* OE efficacy in KOPN-8 B-ALL cells.
(N) Effect of *ZNF217* OE on the growth of KOPN-8 B-ALL cells as determined by MTT assay. Data was presented as mean ± SD (n = 4 biological replicates).
(O) *ZNF217* OE efficacy in SUP-B15 B-ALL cells.
(P) Effect of *ZNF217* OE on the growth of SUP-B15 B-ALL cells as determined by MTT assay. Data was presented as mean ± SD (n = 4 biological replicates).
The p values were calculated using a two-tailed *t*-test. ** $p < 0.01$; *** $p < 0.001$.

Figure S3

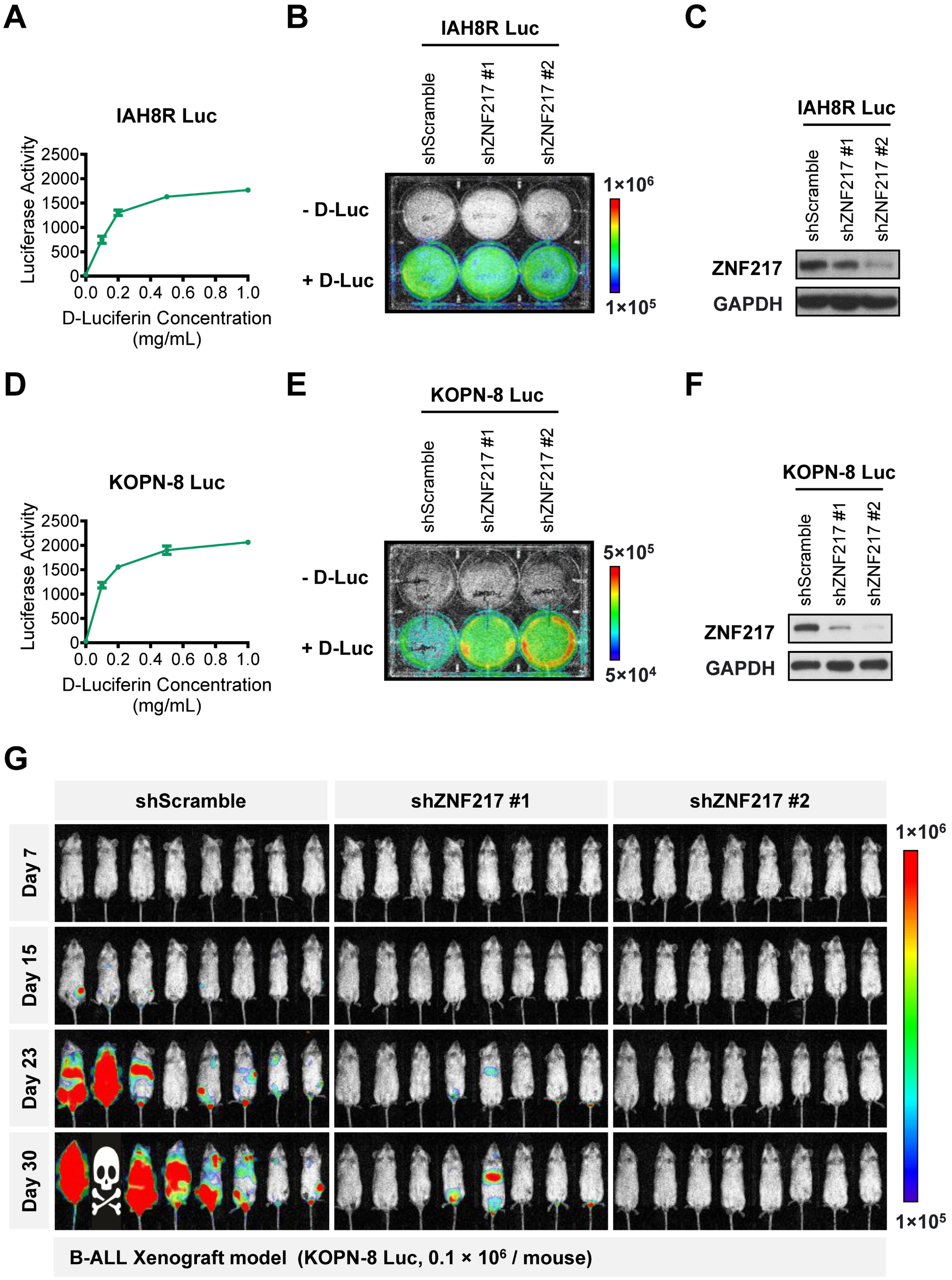

A
IAH8R Luc
Luciferase Activity
2500
2000
1500
1000
500
0
0.0 0.2 0.4 0.6 0.8 1.0
D-Luciferin Concentration (mg/mL)
B
IAH8R Luc
shScramble
shZNF217 #1
shZNF217 #2
- D-Luc
+ D-Luc
1×10⁶
1×10⁵
C
IAH8R Luc
shScramble
shZNF217 #1
shZNF217 #2
ZNF217
GAPDH
D
KOPN-8 Luc
Luciferase Activity
2500
2000
1500
1000
500
0
0.0 0.2 0.4 0.6 0.8 1.0
D-Luciferin Concentration (mg/mL)
E
KOPN-8 Luc
shScramble
shZNF217 #1
shZNF217 #2
- D-Luc
+ D-Luc
5×10⁵
5×10⁴
F
KOPN-8 Luc
shScramble
shZNF217 #1
shZNF217 #2
ZNF217
GAPDH
G
shScramble
shZNF217 #1
shZNF217 #2
Day 7
Day 15
Day 23
Day 30
1×10⁶
1×10⁵
B-ALL Xenograft model (KOPN-8 Luc, 0.1 × 10⁶ / mouse)

Figure S3. The firefly luciferase efficacy in the donor cells of B-ALL PDX and xenograft models, and the effect of *ZNF217* depletion on *in vivo* B-ALL progression. (Related to Figure 3)

(A) The efficacy of firefly luciferase in IAH8R Luc cells. Data was presented as mean ± SD (n = 4 technical replicates).

(B) The efficacy of firefly luciferase in the donor cells of B-ALL PDX model.

(C) *ZNF217* KD efficacy in IAH8R Luc cells.

(D) The efficacy of firefly luciferase in KOPN-8 Luc cells. Data was presented as mean ± SD (n = 4 technical replicates).

(E) The efficacy of firefly luciferase in the donor cells of B-ALL xenograft model.

(F) *ZNF217* KD efficacy in KOPN-8 Luc cells.

(G) Effect of *ZNF217* KD on leukemia burden in B-ALL xenograft model as determined by bioluminescence imaging.

Figure S4

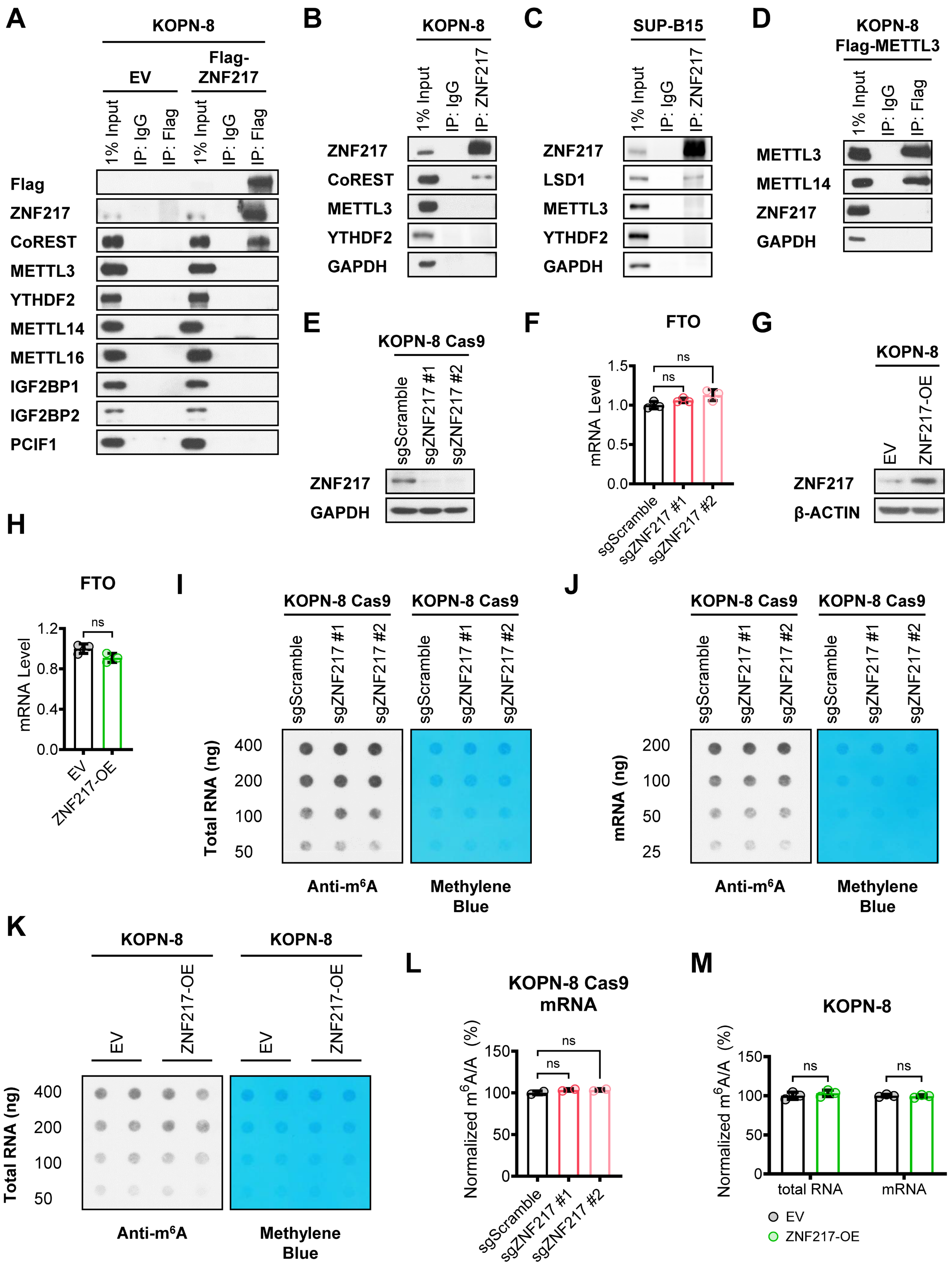

A
KOPN-8
EV
Flag-ZNF217
1% Input
IP: IgG
IP: Flag
Flag
ZNF217
CoREST
METTL3
YTHDF2
METTL14
METTL16
IGF2BP1
IGF2BP2
PCIF1
B
KOPN-8
IP: ZNF217
GAPDH
C
SUP-B15
LSD1
D
KOPN-8 Flag-METTL3
E
KOPN-8 Cas9
sgScramble
sgZNF217 #1
sgZNF217 #2
F
FTO
mRNA Level
ns
G
ZNF217-OE
β-ACTIN
H
I
Total RNA (ng)
Anti-m6A
Methylene Blue
J
mRNA (ng)
K
L
KOPN-8 Cas9 mRNA
Normalized m6A/A (%)
M
total RNA
mRNA
EV
ZNF217-OE

Figure S4. The oncogenic role of ZNF217 in B-ALL is unlikely to be mediated through m^6A-associated mechanisms.

(A) Exogenous Co-IP assay using KOPN-8 cells expressing Flag-tagged ZNF217 or an empty vector.

(B) Endogenous Co-IP assay using KOPN-8 cells.

(C) Endogenous Co-IP assay using SUP-B15 cells.

(D) Exogenous Co-IP assay using KOPN-8 cells expressing Flag-tagged METTL3 or an empty vector.

(E) *ZNF217* KO efficacy in KOPN-8 Cas9 cells.

(F) Effect of *ZNF217* KO on *FTO* transcription in KOPN-8 cell, as determined by RT-qPCR. Data was presented as mean ± SD (n = 3 technical replicates).

(G) *ZNF217* OE efficacy in KOPN-8 Cas9 cells.

(H) Effect of *ZNF217* OE on *FTO* transcription in KOPN-8 cell, as determined by RT-qPCR. Data was presented as mean ± SD (n = 3 technical replicates).

(I) Effect of *ZNF217* KO on the global m^6A level in the total RNA in KOPN-8 cells, as determined by dot blot assay.

(J) Effect of *ZNF217* KO on the global m^6A level in the mRNA in KOPN-8 cells, as determined by dot blot assay.

(K) Effect of *ZNF217* OE on the global m^6A level in the total RNA in KOPN-8 cells, as determined by dot blot assay.

(L) Effect of *ZNF217* KO on the global m^6A level in the mRNA in KOPN-8 cells, as determined by UHPLC-QQQ-MS/MS assay. Data was presented as mean ± SD (n = 2 biological replicates).

(M) Effect of *ZNF217* OE on the global m^6A levels in the total RNA and mRNA in KOPN-8 cells, as determined by UHPLC-QQQ-MS/MS assay. Data was presented as mean ± SD (n = 3 biological replicates).

The p values were calculated using a two-tailed *t*-test. ns, not significant.

Figure S5

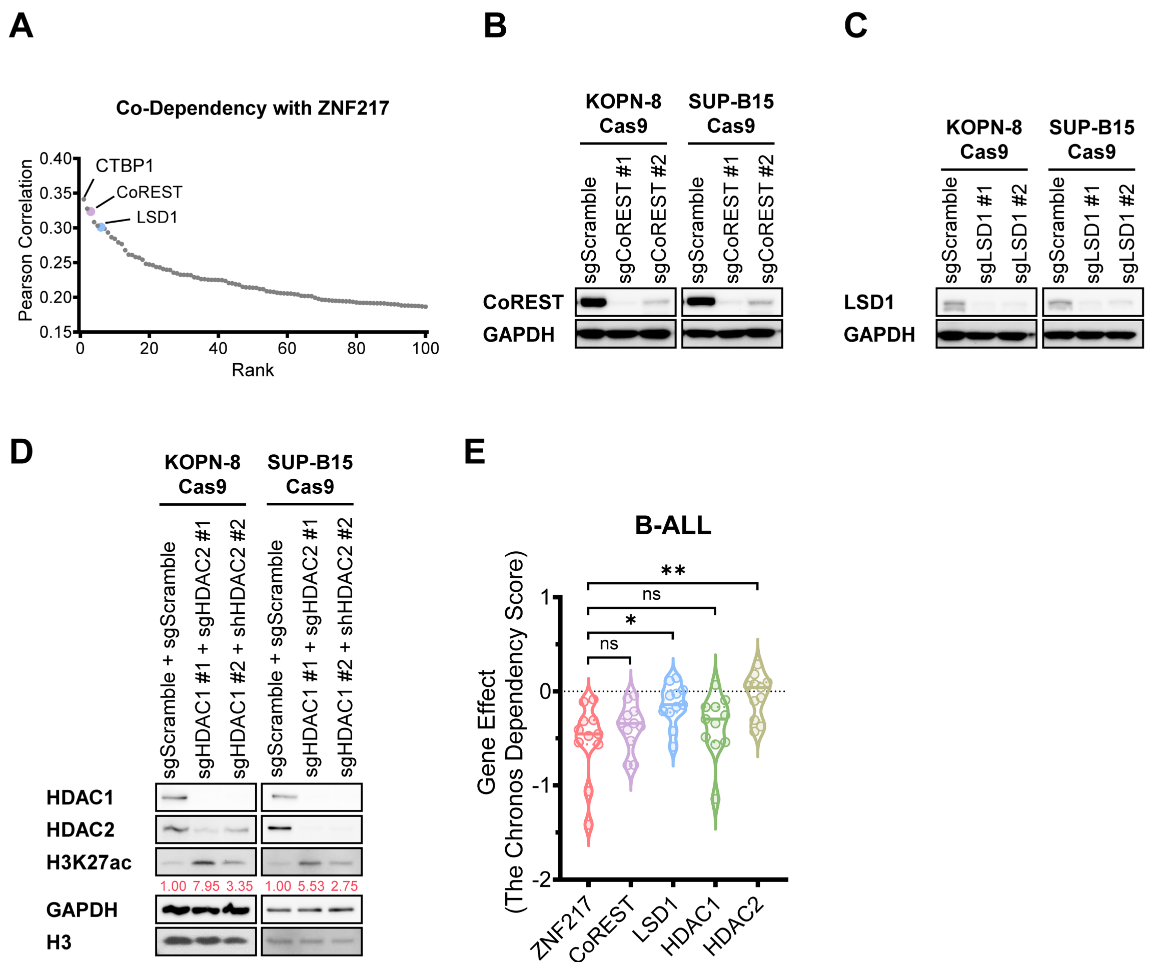

Figure S5. ZNF217 collaborates with the CoREST complex to regulate histone modifications. (Related to Figure 4)

(A) The top 100 genes exhibiting co-dependency with *ZNF217* across 1,139 cancer cell lines. Data was retrieved from the DepMap portal. Co-dependency rankings are based on Pearson correlation coefficients between the genes' corresponding dependency scores.

(B) *CoREST* KO efficiency in KOPN-8 Cas9 and SUP-B15 Cas9 cells as determined by western blotting.

(C) *LSD1* KO efficiency in KOPN-8 Cas9 and SUP-B15 Cas9 cells as determined by western blotting.

(D) *HDAC1/HDAC2* KO efficiency in KOPN-8 Cas9 and the effect of *HDAC1/HDAC2* KO on H3K27ac deacetylation as determined by Western blotting. Quantification of H3K27ac levels is shown in red beneath the corresponding Western blot bands.

(E) The Chronos Dependency Scores of ZNF217, CoREST, LSD1, HDAC1, and HDAC2 in B-ALL cell lines. All the Chronos Dependency Scores were derived from the DepMap portal (https://depmap.org/portal/). n = 11 for each gene.

The p values were calculated using a two-tailed t-test. ns, not significant; * $p < 0.05$; ** $p < 0.01$.

Figure S6

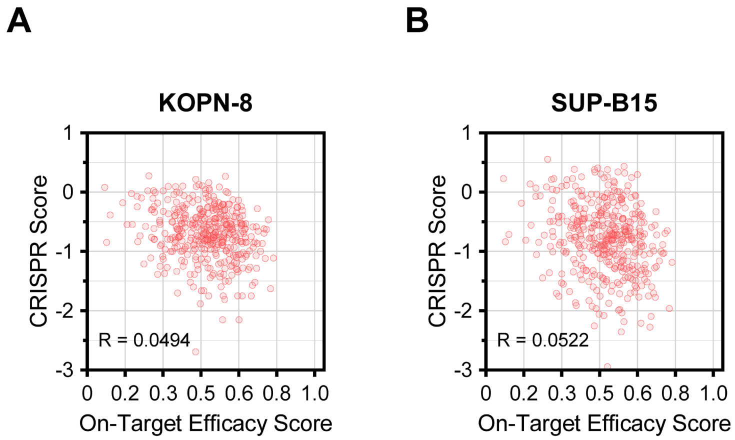

Figure S6. Correlations between the CRISPR scores of ZNF217 sgRNAs and their on-target efficacy scores. (Related to Figure 5)

(A) Correlation between the CRISPR scores of 416 *ZNF217* sgRNAs in KOPN-8 Cas9 single clones and their estimated on-target efficacy scores.

(B) Correlation between the CRISPR scores of 416 *ZNF217* sgRNAs in SUP-B15 Cas9 single clones and their estimated on-target efficacy scores.

On-target efficacy scores were predicted using the Genetic Perturbation Platform (https://portals.broadinstitute.org/gpp/public/).

Figure S7

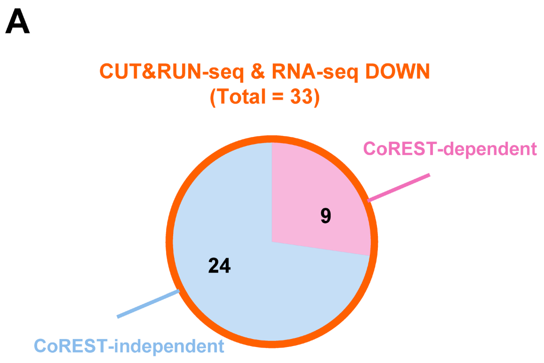

Figure S7. Multiple-omics analysis of ZNF217 downstream targets in B-ALL cells (Related to Figure 6)

(A) The overlap of downregulated genes identified by RNA-seq and ZNF217-bound genes identified by CUT&RUN-seq.

Figure S8

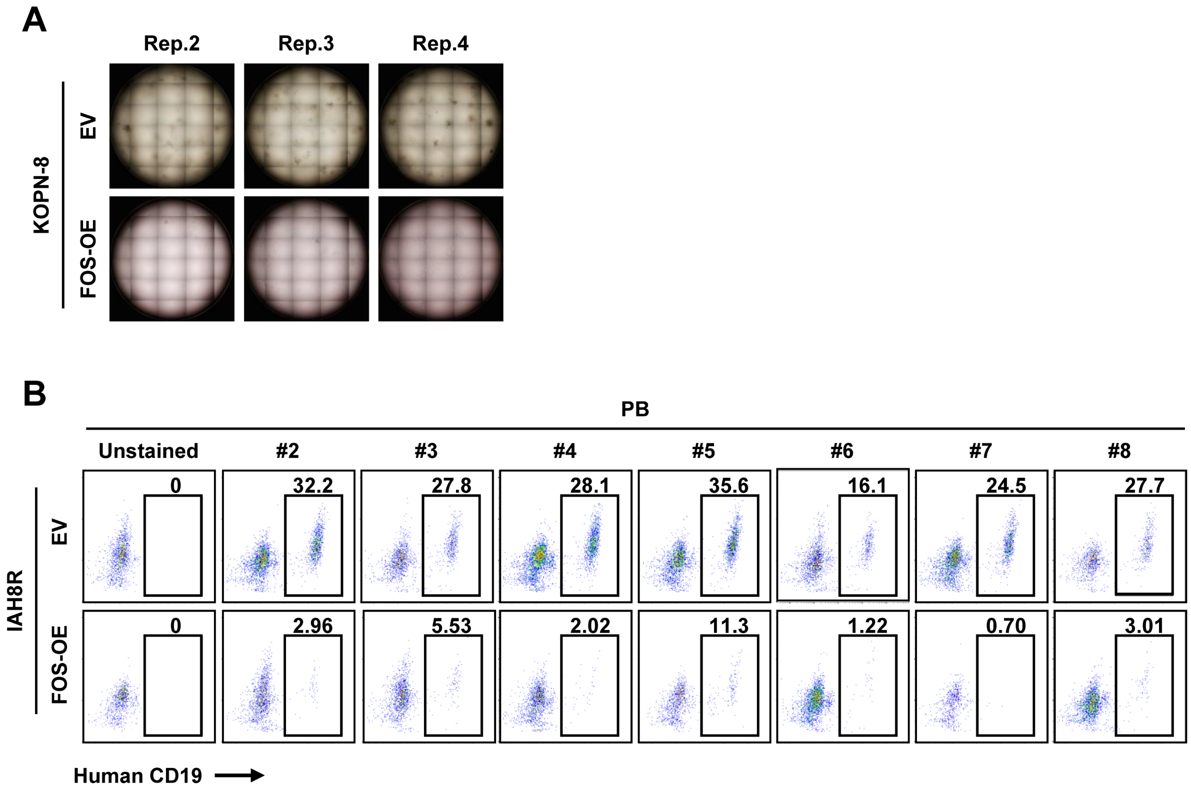

Figure S8. FOS plays a tumor suppressor role in B-ALL. (Related to Figure 7)
(A) Effect of *FOS* OE on the colony-forming ability of KOPN-8.
(B) Effect of *FOS* OE on leukemia burden in B-ALL PDX recipient (NSG) mice, as determined by flow cytometry measuring the percentage of human CD19$^+$ cells in the peripheral blood of recipient mice.
